# Activated Brain Endothelial Cells Cross-Present Malaria Antigen

**DOI:** 10.1371/journal.ppat.1004963

**Published:** 2015-06-05

**Authors:** Shanshan W. Howland, Chek Meng Poh, Laurent Rénia

**Affiliations:** 1 Singapore Immunology Network, Agency for Science, Technology and Research (A*STAR), Singapore; 2 Department of Microbiology, Yong Loo Lin School of Medicine, National University of Singapore, Singapore; Francis Crick Institute, UNITED KINGDOM

## Abstract

In the murine model of cerebral malaria caused by *P*. *berghei* ANKA (PbA), parasite-specific CD8^+^ T cells directly induce pathology and have long been hypothesized to kill brain endothelial cells that have internalized PbA antigen. We previously reported that brain microvessel fragments from infected mice cross-present PbA epitopes, using reporter cells transduced with epitope-specific T cell receptors. Here, we confirm that endothelial cells are the population responsible for cross-presentation *in vivo*, not pericytes or microglia. PbA antigen cross-presentation by primary brain endothelial cells *in vitro* confers susceptibility to killing by CD8^+^ T cells from infected mice. IFNγ stimulation is required for brain endothelial cross-presentation *in vivo* and *in vitro*, which occurs by a proteasome- and TAP-dependent mechanism. Parasite strains that do not induce cerebral malaria were phagocytosed and cross-presented less efficiently than PbA *in vitro*. The main source of antigen appears to be free merozoites, which were avidly phagocytosed. A human brain endothelial cell line also phagocytosed *P*. *falciparum* merozoites. Besides being the first demonstration of cross-presentation by brain endothelial cells, our results suggest that interfering with merozoite phagocytosis or antigen processing may be effective strategies for cerebral malaria intervention.

## Author Summary

Cerebral malaria accounts for most of the deaths caused by *Plasmodium* infection. In the mouse model of cerebral malaria, CD8^+^ T cells are known to be the effector cells responsible for lethal neuropathology, but it was not clear how they disrupted the blood-brain barrier. Here, we show that brain endothelial cells cross-present parasite antigen at the onset of pathology, hence allowing recognition by parasite-specific cytotoxic T lymphocytes. This process did not occur in mice lacking IFNγ, whereas TNFα and LTα were dispensable. The proposed mechanism of pathogenesis was recapitulated *in vitro*: IFNγ-stimulated primary mouse brain endothelial cells cultured with parasite-infected red blood cells were subsequently killed by CD8^+^ T cells isolated from an infected mouse. The murine endothelial cells primarily phagocytose not infected red blood cells but instead merozoites, the form of the parasite that infects red blood cells. We show also that human brain endothelial cells phagocytose *P*. *falciparum* merozoites *in vitro*, suggesting that our findings with the mouse model may be relevant to human disease. Strategies to interfere with merozoite phagocytosis or antigen processing by endothelial cells may prove useful for treating cerebral malaria.

## Introduction

Half the world’s population is at risk of malaria infection, which is estimated to kill half a million children under the age of 5 annually [1]. Most of these fatalities occur due to a severe complication of *Plasmodium falciparum* (Pf) infection called cerebral malaria, with clinical features of impaired consciousness, seizures and abnormal posturing. Autopsies frequently reveal brain swelling and petechial hemorrhages, and most characteristically, dense sequestration of parasitized red blood cells in many brain microvessels [2]. Mechanistic understanding of the etiology of cerebral malaria remains elusive, given the ethical limitations of research in human patients. The mouse model of experimental cerebral malaria (ECM) induced by *P*. *berghei* ANKA (PbA) infection recapitulates many features of the human disease including parasite accumulation in the brain, albeit controversially to a much less prominent degree [3]. Extensive evidence has emerged that ECM is an immune-mediated disease, with roles described for CD4^+^ and CD8^+^ T cells [4–6], γδ T cells [7], NK cells [8], NKT cells [9], neutrophils [10], monocytes [11], microglia [12], and splenic CD8^+^ dendritic cells [13,14]. Amongst these cell types, CD8^+^ T cells play a unique effector role in ECM pathogenesis as their depletion one day before neurological symptoms are expected prevents disease [5]. In contrast, CD4^+^ T cells [5], γδ T cells [7] and neutrophils [10] have to be depleted early to be efficacious, and NK cells and CD4^+^ T cells in particular were found to act by recruiting CD8^+^ T cells to the brain via IFNγ [8,15,16]. Adoptive transfer experiments revealed that the pathogenicity of CD8^+^ T cells was dependent on perforin and Granzyme B expression [6,17], suggesting that their cytolytic function was directly responsible for the loss of blood-brain barrier integrity observed in ECM.

In the past few years, we and others have identified a number of PbA blood-stage epitopes, confirming the pathogenic role of antigen-specific CD8^+^ T cells in ECM [18–21]. By transferring TCR-transgenic CD8^+^ T cells (PbT-I T cells recognizing the PbA epitope NCYDFNNI) into hosts depleted of endogenous CD8^+^ T cells, Lau *et al*. showed definitively that PbA-specific CD8^+^ T cells can induce lethal neurological damage [20]. However, several of the known blood-stage epitopes are also conserved in other rodent malaria species or strains that do not cause ECM, and we found to our surprise that cytotoxic CD8^+^ T cells specific to these epitopes are also induced during these infections [19,21]. We asked whether the difference between PbA and non-ECM-causing strains could lie in the prevalence of targets for CD8^+^ T cell-mediated cytolysis amongst the cells constituting the blood-brain barrier i.e. cells that were cross-presenting parasite antigens. We first addressed this question using NFAT-lacZ reporter cells transduced with a TCR recognizing the Pb1 epitope (SQLLNAKYL, from the GAP50 protein), which turn blue following X-gal staining if they encounter Pb1 in the context of H-2D^b^ MHC class I molecules [19]. These LR-BSL8.4a reporter cells were incubated with brain microvessel fragments isolated from naïve mice and infected mice. Only brain microvessels from PbA-infected mice, but not those from mice infected with non-ECM-causing *P*. *berghei* NK65 (PbNK65) or *P*. *yoelii* 17XNL (Py17X), gave rise to elevated numbers of blue reporter cells. Further experiments with reporter cells recognizing two other epitopes gave similar results—only PbA infection led to brain microvessel cross-presentation of parasite antigen, supporting the proposition that such cross-presentation is a necessary step in ECM pathogenesis [21]. Another indication that CD8^+^ T cells need to act on cross-presenting brain microvessel cells to cause ECM comes from experiments in which mice were rescued from ECM by treatment with anti-malarial drugs one day before symptoms were expected. Compared to untreated mice, the treated mice had similar numbers of antigen-specific brain-sequestered CD8^+^ T cells, but brain microvessel cross-presentation was severely reduced [19].

The importance of proinflammatory cytokines in ECM pathogenesis has been a topic of considerable interest. A crucial role for IFNγ was demonstrated using mice deficient for either the cytokine [4] or its receptor [22]. The association between TNFα and human cerebral malaria and ECM has been extensively studied, but Engwerda *et al*. showed that TNFα knockout (KO) mice were susceptible to ECM while LTα KO mice were protected [23]. Here, we first investigate the cytokine requirements for cross-presentation during ECM and identify the cell type involved, then develop an *in vitro* PbA cross-presentation model to gain mechanistic insight.

## Results

### IFNγ is required for brain microvessel cross-presentation

**Fig 1:**
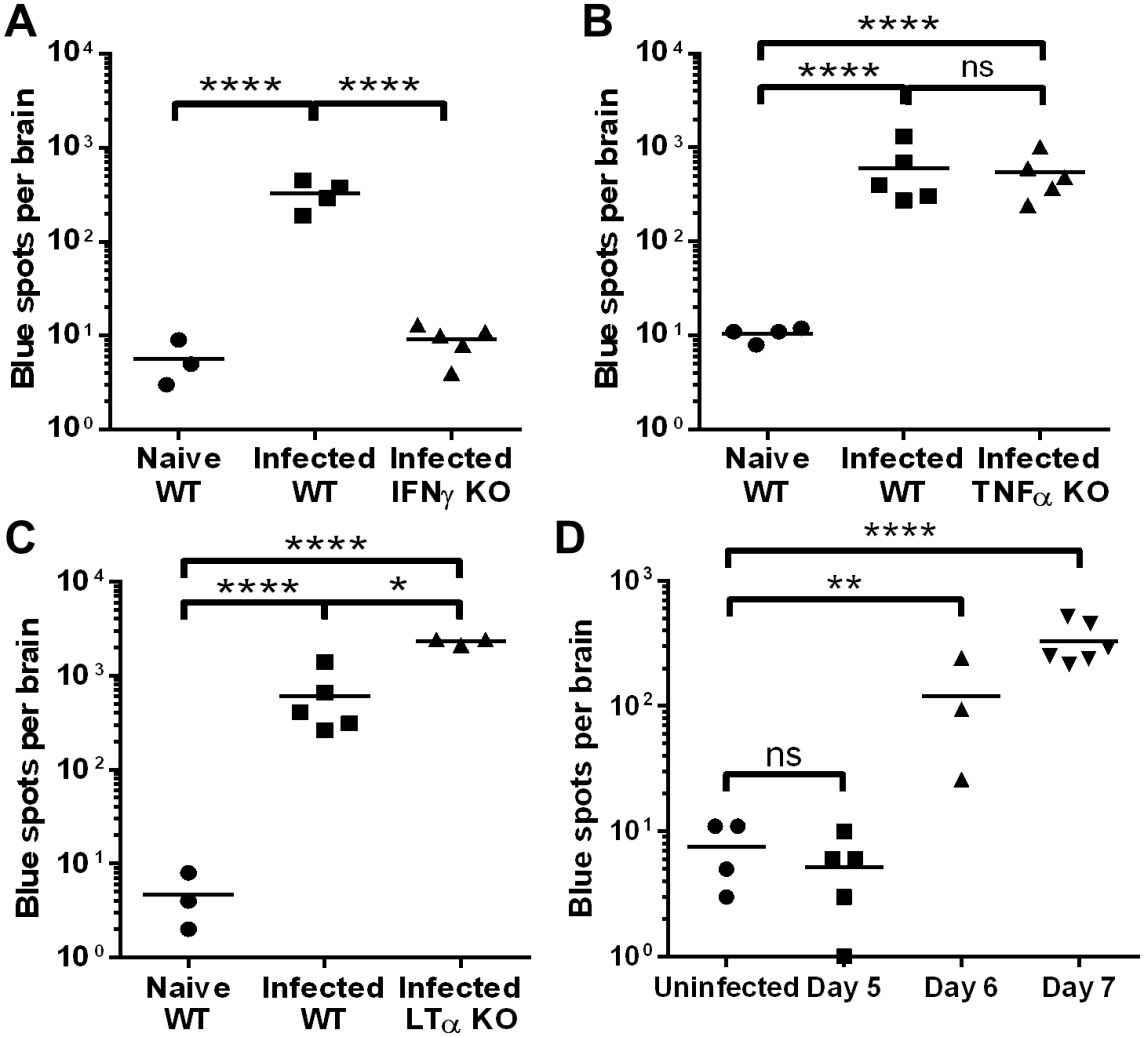
Cytokine requirements and timing of brain microvessel cross-presentation during PbA infection. (A–C) WT mice and mice deficient for IFNγ (A), TNFα (B), and LTα (C) were infected with PbA. After 7 days, brain microvessels were isolated from the infected mice as well as naïve WT mice, and incubated overnight with LR-BSL8.4a reporter cells. The total number of blue cells (counted as blue spots by the ELISOT reader) resulting from each brain after X-gal staining was quantified. *P<0.05, ****P<0.0001, ns not significant, ANOVA with Bonferroni’s post-test on log-transformed numbers. (D) Brain microvessels from uninfected mice and WT mice infected with PbA 5, 6, or 7 days previously were tested for cross-presentation as above. **P<0.01, ****P<0.0001, ns not significant as compared to uninfected mice by ANOVA with Bonferroni’s post-test on log-transformed numbers.

We examined the roles of IFNγ, TNFα and LTα in brain microvessel cross-presentation during PbA infection by comparing mice deficient in each cytokine with wild type (WT) C57BL/6J mice. Brain microvessel fragments were isolated from infected and naïve mice and incubated overnight with the reporter cell line, LR-BSL8.4a, to measure Pb1 presentation. Brain microvessels from IFNγ KO mice yielded background numbers of blue spots (each corresponding to a responding reporter cell), showing that IFNγ is necessary for cross-presentation in the brain (Fig 1A). In contrast, both TNFα and LTα are dispensable for brain microvessel cross-presentation as the assay readouts in the knockout mice were not significantly different or even higher than in WT mice (Fig 1B and 1C). The higher cross-presentation levels in LTα KO mice may indicate impaired CD8^+^ T cell-mediated killing of cross-presenting cells or increased survival of the latter. We also investigated the timing of brain microvessel cross-presentation in PbA-infected WT mice. Cross-presentation was not observed 5 days post-infection, started to occur at intermediate levels on day 6 and reached high levels on day 7, when ECM signs typically emerge (Fig 1D).

### Identification of cross-presenting cell types

In our earlier work, we were unable to identify the brain microvessel cell type that was responsible for cross-presentation, because of the difficulty in obtaining a single cell suspension of viable cells with collagenase digestion [19]. However, we noted that completely eliminating collagenase treatment led to much lower activation of reporter cells, suggesting that the cross-presenting cells may be endothelial cells or pericytes that are surrounded by collagen-rich basal lamina. We have continued to experiment with different digestion enzymes and protocols to allow brain microvessel cells to be sorted. Digestion of microvessels with Liberase DH (Roche) yielded viable single cells that could be separated by FACS, but none of the sorted populations were able to activate LR-BSL8.4a reporter cells.

**Fig 2:**
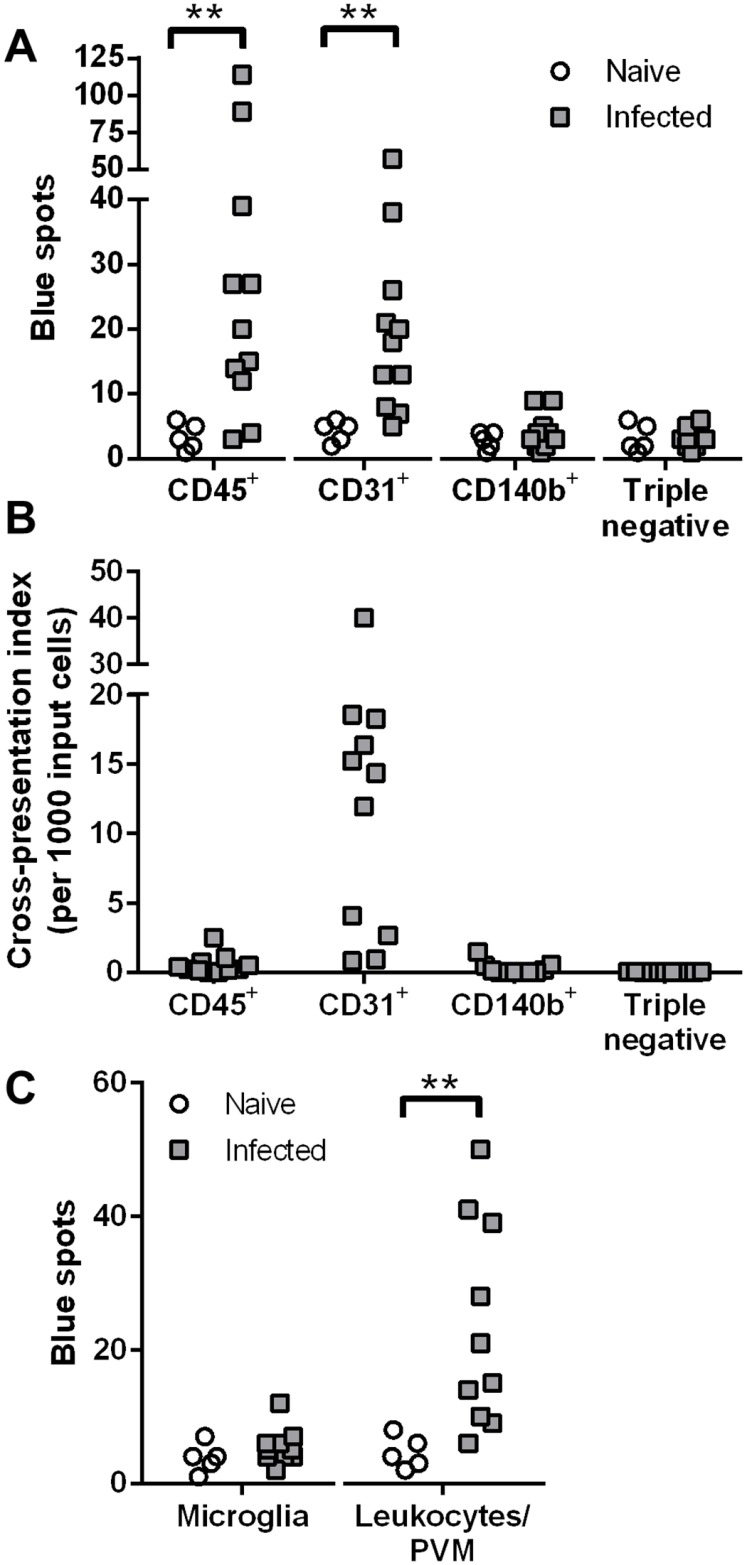
Endothelial cells and leukocytes are the cross-presenting cells in the brain during PbA infection. (A–B) Brains from naïve (*n* = 5) and PbA-infected mice exhibiting signs of ECM (*n* = 11) were subjected to automated dissociation using papain. After myelin removal, the single cell suspensions were stained and sorted into four populations: CD45^+^ microglia and leukocytes, CD45^-^CD31^+^ endothelial cells, CD45^-^CD140b^+^ cells including pericytes, and cells negative for all three markers. Each sorted population was tested for cross-presentation by incubating with LR-BSL8.4a cells and staining with X-gal. Data were pooled from 3 independent experiments. (A) Raw spot counts. **P< 0.01, separate Mann-Whitney U tests comparing naïve vs infected for each population. (B) Only data from infected mice are shown, after background subtraction (the mean of all naïve wells) and normalization by the number of sorted cells added. Negative data are plotted as zeroes. (C) Brains from naïve (*n* = 5) and PbA-infected mice (*n* = 10) were manually dissociated and digested with collagenase. After myelin removal, the single cell suspensions were stained and sorted for microglia (CD45^int^CD11b^+^) and peripheral leukocytes together with PVM (CD45^hi^), which were assayed for cross-presentation as above. Data were pooled from 2 independent experiments. **P< 0.01, Mann-Whitney U test. See S1 Fig for representative FACS plots.

Ultimately, we succeeded using a papain-based Neural Tissue Dissociation Kit (Miltenyi) with automated mechanical dissociation of the entire brain. Single cell suspensions from naïve and PbA-infected mouse brains were separated into four populations after antibody labeling. First, all CD45^+^ cells (microglia and leukocytes) were gated, then the CD45^-^ cells were further subdivided into CD31^+^ endothelial cells, a CD140b^+^ population including pericytes, and triple negative cells such as astrocytes and neurons (S1A Fig). After overnight incubation with LR-BSL8.4a reporter cells and X-gal staining, only the CD45^+^ and CD31^+^ populations gave rise to elevated numbers of blue spots comparing infected mice to naïve mice (Fig 2A). The numbers of blue spots induced by sorted endothelial cells were much smaller than those typically arising from brain microvessel fragments (tens compared to hundreds). One reason could be the low yield of endothelial cells with the Neural Tissue Dissociation Kit, averaging 1.7 × 10^3^ cells sorted per brain compared to 1.6 × 10^4^ cells from Liberase digestion of isolated brain microvessels. Further, while papain was selected because it largely preserves CD31 staining, its ability to cleave the heavy chain of MHC class I molecules is well-known. Despite reducing the papain concentration, it is possible that we were only able to detect “new” Pb1-H-2D^b^ complexes that were generated *in vitro* from intracellular antigen stores. Because endothelial cells were isolated with much lower efficiency than leukocytes, we have also plotted the numbers of blue spots normalized by the input cell number, making it evident that endothelial cells were by far the most efficient cross-presenters on a per-cell basis (Fig 2B).

Since the CD45^+^ population also gave a positive signal, we wished to clarify whether microglia were responsible. The ability of microglial cells to cross-present soluble antigens has recently been demonstrated *in vivo* [24], and evidence of malaria antigen cross-presentation by these cells would have important implications. In the follow-up experiments, brains were digested with collagenase (papain digestion being unnecessary and disadvantageous as noted above) for sorting into CD45^int^CD11b^+^ microglia and CD45^hi^ peripheral leukocytes and perivascular macrophages (PVM) as shown in S1B Fig. Cross-presentation of the Pb1 epitope was detected from the CD45^hi^ fraction but not from microglia from infected mice (Fig 2C). Thus, while microglial cells have the potential to cross-present, they do not do so appreciably during PbA infection, perhaps because of limited access to antigen.

### An *in vitro* model of endothelial PbA cross-presentation

**Fig 3:**
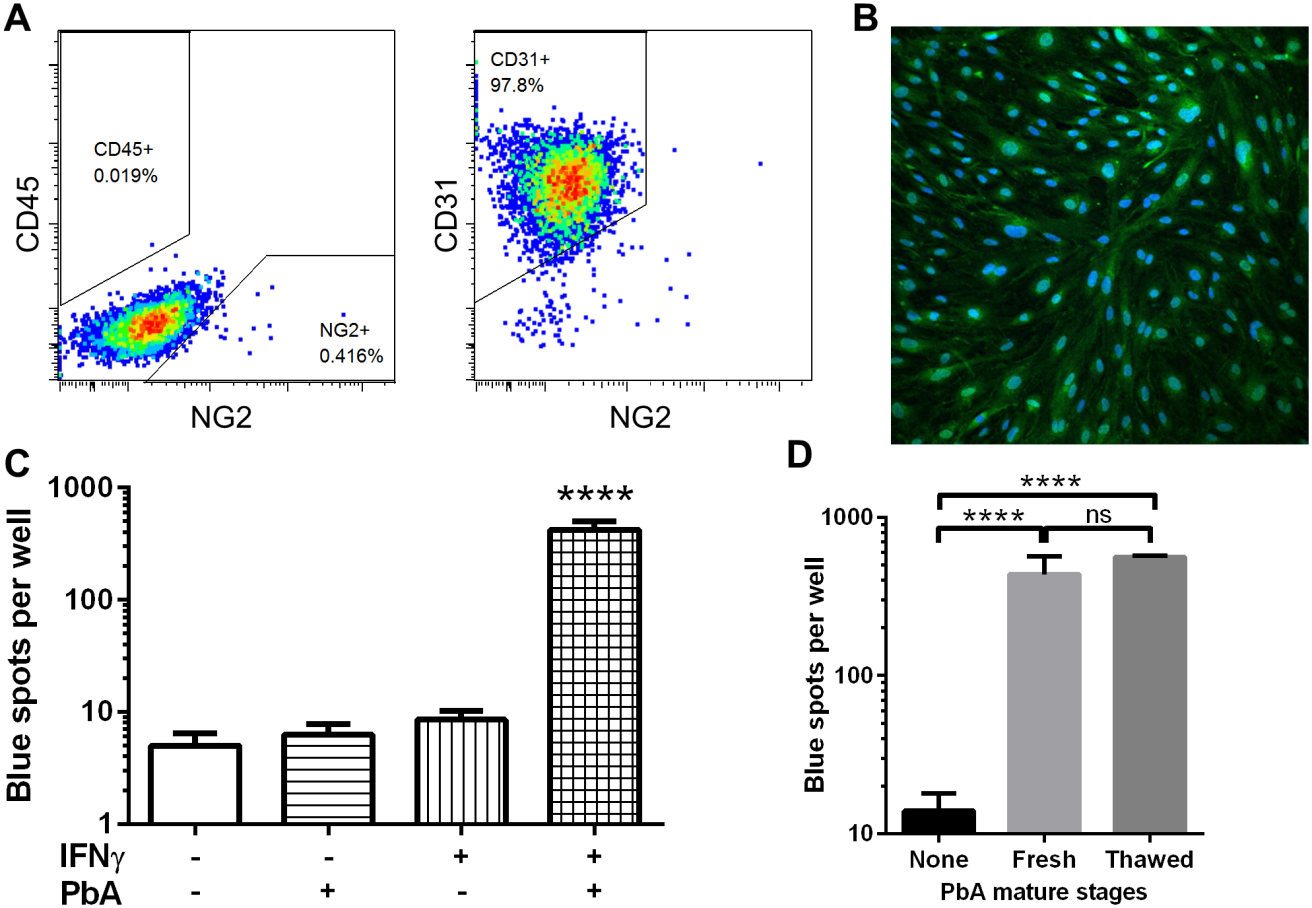
IFNγ-stimulated MBECs cross-present PbA antigen *in vitro*. (A) After culturing mouse brain microvessels for 10 days including puromycin selection, the resulting cells are >97% CD45^-^CD31^+^ endothelial cells. Dot plots are representative of 3 independent culture experiments. (B) MBECs were fixed and stained for the endothelial marker von Willebrand factor (green) and counterstained with DAPI (blue). (C) MBECs in quadruplicate wells of a 48-well plate were stimulated (or not) with 10 ng/ml IFNγ for 24 h, then 5 × 10^6^ thawed PbA mature iRBCs were added (or not) for 24 h. The wells were washed and co-cultured with LR-BSL8.4a reporter cells overnight prior to X-gal staining. ****P<0.0001 compared to every other group, ANOVA with Bonferroni’s post test on log-transformed blue spot counts. (D) Cross-presentation by IFNγ-stimulated MBECs was similarly assayed after pulsing with 10^6^ freshly isolated or freeze-thawed PbA mature stages (*n* = 4). ****P< 0.0001, ns not significant, ANOVA with Bonferroni’s post test on log-transformed blue spot counts.

To further understand how brain endothelial cells cross-present during ECM, we sought to recapitulate the process *in vitro* using primary cultures of murine brain endothelial cells (MBECs). Brain microvessels isolated from naïve mice were cultured on collagen-coated plates using puromycin to kill non-endothelial cells for the first 2–3 days [25]. In our hands, after 10 days of culture, >97% of the cells were CD31^+^ endothelial cells, with essentially no CD45^+^ cell contamination and <0.5% NG2^+^ pericyte contamination (Fig 3A). The cultures also expressed the endothelial marker von Willebrand factor (Fig 3B). We always used unpassaged primary MBEC cultures to retain the original phenotype as much as possible. Unstimulated MBECs and IFNγ-stimulated MBECs were incubated with frozen-thawed PbA mature iRBCs (late trophozoites and schizonts) for 24 h, after which they were washed and co-incubated with LR-BSL8.4a reporter cells overnight. After X-gal staining of the reporter cells, only MBECs that had been exposed to both IFNγ and PbA-infected iRBCs were found to have cross-presented (Fig 3C). Both the ability of endothelial cells to cross-present PbA antigen and the necessity of IFNγ stimulation agree with our *ex vivo* results from infected mice. Similar results were obtained with reporter cell lines that we created to detect cross-presentation of two additional PbA epitopes (S2A and S2B Fig). Frozen mature iRBCs were used as antigen in these and many subsequent experiments for convenience; we compared these to freshly isolated PbA mature iRBCs and found no significant difference in cross-presentation efficiency (Fig 3D). We also confirmed that uninfected RBCs gave readouts indistinguishable from the background (S3A Fig).

Neither TNFα nor LTα were able to induce MBEC cross-presentation, nor could they enhance cross-presentation when used in conjunction with IFNγ (S2C Fig). The ability of MBECs to cross-present appears to be general rather than specific to malaria antigen: cross-presentation of soluble ovalbumin occurred even without IFNγ stimulation but was enhanced by it (S2D Fig). Wheway *et al*. recently showed that a human brain endothelial cell line expressed high basal levels of MHC class I not further enhanced by IFNγ [26]. In contrast, we found MHC class I expression on MBECs to be highly IFNγ-inducible (S2E Fig), which may partly explain the importance of this cytokine in cross-presentation. In addition, IFNγ upregulated MBEC expression of ICAM-1 but not VCAM-1 (S2E Fig).

### CD8^+^ T cells induced during infection can kill cross-presenting MBECs

**Fig 4:**
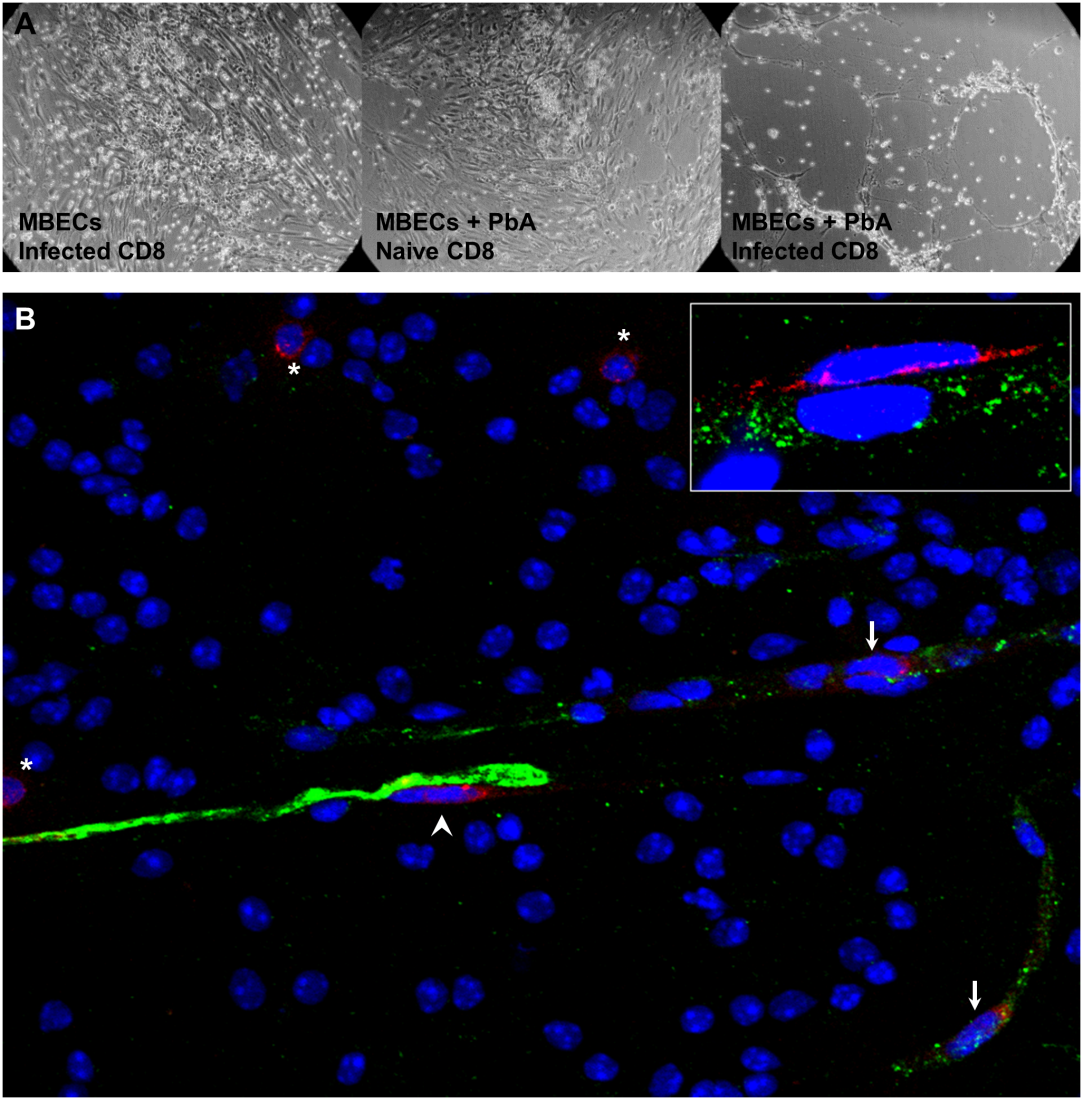
Interactions between CD8^+^ T cells and MBECs. (A) IFNγ-stimulated MBECs were incubated with (or without) 3 × 10^6^ thawed PbA mature iRBCs for 24 h. After washing, we added 10^6^ CD8^+^ T cells from either a naïve mouse or one infected with PbA 6 days previously. The wells were washed gently and photographed (DIC) after 20 h. Images are representative of triplicate wells. (B) An olfactory bulb smear from a mouse with ECM was fixed and stained with antibodies against von Willebrand factor (green) and CD8 (red). CD8^+^ T cells were present within blood vessels (arrows), on the abluminal face of endothelial cells (arrow head) and in the parenchyma (asterisks), 40× objective. Inset: a perivascular CD8^+^ T cell in close contact with the endothelium (100× objective).

The prevailing model of ECM pathogenesis attributes severe disruption of the blood-brain barrier to cytolysis of cross-presenting endothelial cells by PbA-specific cytotoxic T lymphocytes. To test this model, instead of adding reporter cells to IFNγ-stimulated MBECs pulsed with PbA mature iRBCs, we added CD8^+^ T cells isolated from a PbA-infected mouse just prior to ECM. The MBECs were indeed almost entirely dead after 20 h of co-incubation (Fig 4A). When PbA antigen was omitted, the activated CD8^+^ T cells adhered to the endothelial cells but did not kill them. When CD8^+^ T cells from a naïve mouse were added to PbA-pulsed MBECs, the endothelial monolayer also remained largely intact, although there may have been some MBEC dysfunction caused by interaction with parasite molecules (reviewed by Razakandrainibe et al. [27]). Hence, this *in vitro* experiment confirms that MBECs cross-presenting PbA antigen can be recognized for killing by the parasite-specific CD8^+^ T cell response.

To observe interactions between CD8^+^ T cells and endothelial cells *in situ*, we performed immunofluorescence staining of olfactory bulb smears. The olfactory bulb is the site of early and severe damage in ECM [28]. Brain smears allow the endothelial cells to be visualized more efficiently compared to tissue sections cutting through blood vessels at random planes. In mice with ECM, CD8^+^ T cells were found not only within blood vessels, but also on the abluminal face of endothelial cells and in the parenchyma (Fig 4B). The perivascular CD8^+^ T cells were flattened and in intimate contact with the endothelial cells (Fig 4B inset), indicating cell-cell interaction. No CD8^+^ T cells were observed in olfactory bulb smears from naïve mice. It has to be kept in mind that during ECM, the number of CD8^+^ T cells for a whole mouse brain is less than 100 000, of which only a fraction may be specific for parasite antigens. Thus, the few CD8^+^ T cells observed in the smears are consistent with the paucity of these cells in the brain of mice with ECM. In addition, the strong killing as seen *in vitro* with high CD8^+^ T cell to endothelial cell ratio is unlikely to be observed *in vivo*.

### MBEC cross-presentation occurs by the cytosolic route

**Fig 5:**
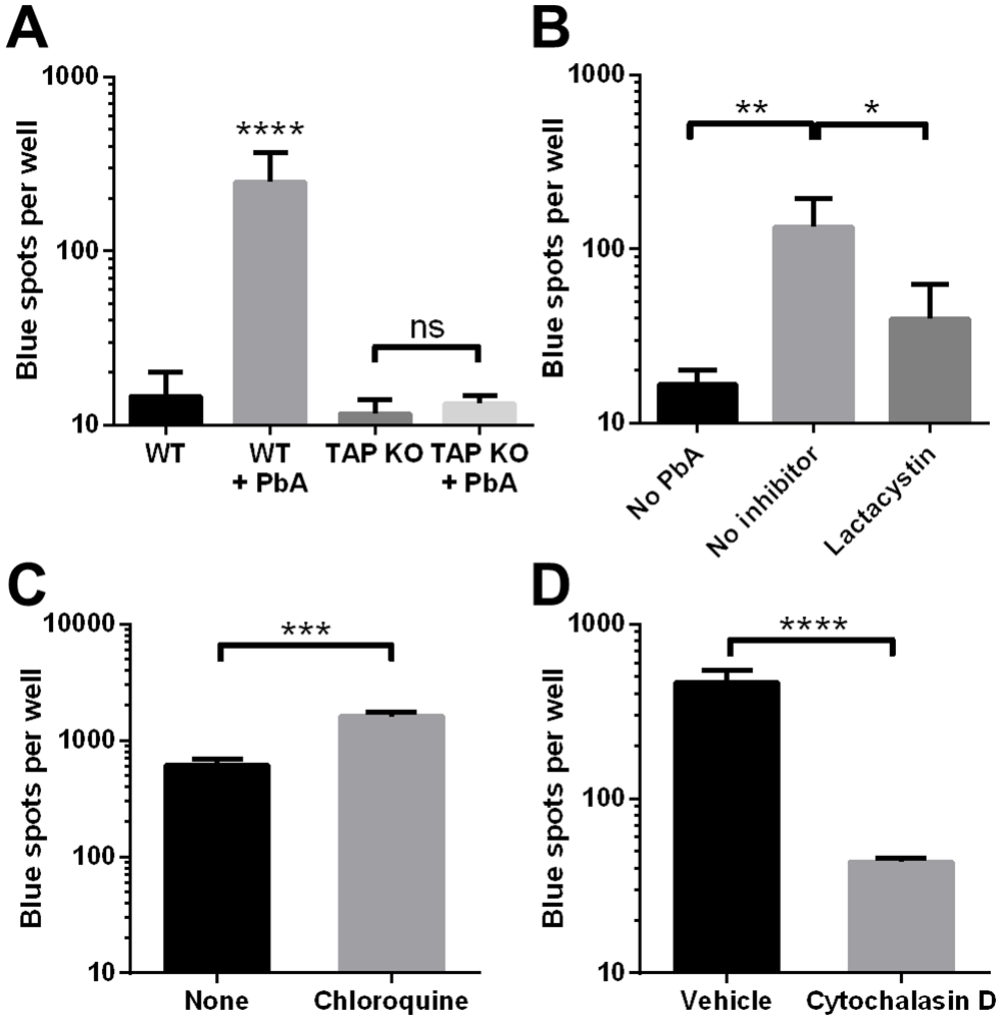
Cross-presentation of PbA antigen by MBECs occurs by the cytosolic pathway. (A) MBEC cultures were established from WT mice as well as TAP1-deficient mice and stimulated with IFNγ. PbA mature iRBCs were added to some wells in triplicate. After 24 h, the wells were washed and cross-presentation of Pb1 was detected using LR-BSL8.4a cells and X-gal staining. ****P<0.0001, ns not significant, ANOVA with Bonferroni’s post test on log-transformed data. Results are representative of 2 independent experiments. (B) PbA mature iRBCs were added (or not) to IFNγ-stimulated MBECs. Lactacystin (10 μM) was added to some wells 6 h later. After another 6 h, all wells were washed and assayed for Pb1 cross-presentation using LR-BSL8.4a cells. *n* = 3, *P<0.05, **P<0.01, ANOVA with Bonferroni’s post test on log-transformed data. Results are representative of 2 independent experiments. (C, D) PbA mature iRBCs were added to IFNγ-stimulated MBECs 1 h after either 10 μg/ml chloroquine diphosphate (C) or 10 μM cytochalasin D (D) were added to some wells. The MBECs were washed and co-incubated with LR-BSL8.4a cells 24 h later to measure Pb1 cross-presentation. *n* = 3, ***P<0.001, ****P<0.0001, unpaired t-test on log-transformed spot counts.

During cross-presentation, the peptides to be loaded onto MHC class I molecules may either be generated by the proteasome for subsequent transportation by TAP (the cytosolic route) or by endolysosomal proteases (the TAP-independent, vacuolar route). To investigate the molecular mechanism of MBEC cross-presentation of PbA antigen, we first cultured MBECs from TAP1-deficient mice. In contrast to WT MBECs, MBECs lacking TAP1 were completely unable to activate LR-BSL8.4a cells after IFNγ stimulation and exposure to PbA (Fig 5A). We used the proteasome inhibitor lactacystin to study the role of proteasomes. We encountered some technical difficulty performing the experiment because prolonged lactacystin exposure was toxic to the MBECs, while cross-presentation required extended co-incubation with PbA mature iRBCs. We found that 6 h of exposure to 10 μM lactacystin did not affect the viability or MHC class I expression level of MBECs, as assessed by their ability to activate LR-BSL8.4a cells after pulsing with Pb1 peptide (S2F Fig). MBECs were therefore incubated with PbA mature stages for 12 h, with lactacystin present during the second half of this time, before the wells were washed and assayed for Pb1 cross-presentation. The number of blue spots obtained from lactacystin-treated wells was significantly reduced (by about 70%) compared to wells without inhibitor, revealing the proteasome-dependence of cross-presentation (Fig 5B). Conversely, chloroquine, an inhibitor of endosome acidification and thus the vacuolar route, did not reduce cross-presentation efficiency when present throughout the 24 h incubation with PbA antigen (Fig 5C). Instead, the number of blue reporter cells increased significantly, suggesting that endolysosomal proteases destroyed rather than generated the MHC class I epitope. MBEC cross-presentation of PbA mature iRBCs was sensitive to cytochalasin D (Fig 5D), consistent with but not conclusive for antigen uptake by phagocytosis. No cross-presentation was seen when PbA mature stages were separated from MBECs by a Transwell support with 0.4 μm pores, arguing against soluble proteins being the source of cross-presented antigen (S3B Fig). Taken together, the evidence points towards PbA cross-presentation by the phagosome-to-cytosol route, requiring proteasomes and TAP.

### Merozoites are the primary antigen source

**Fig 6:**
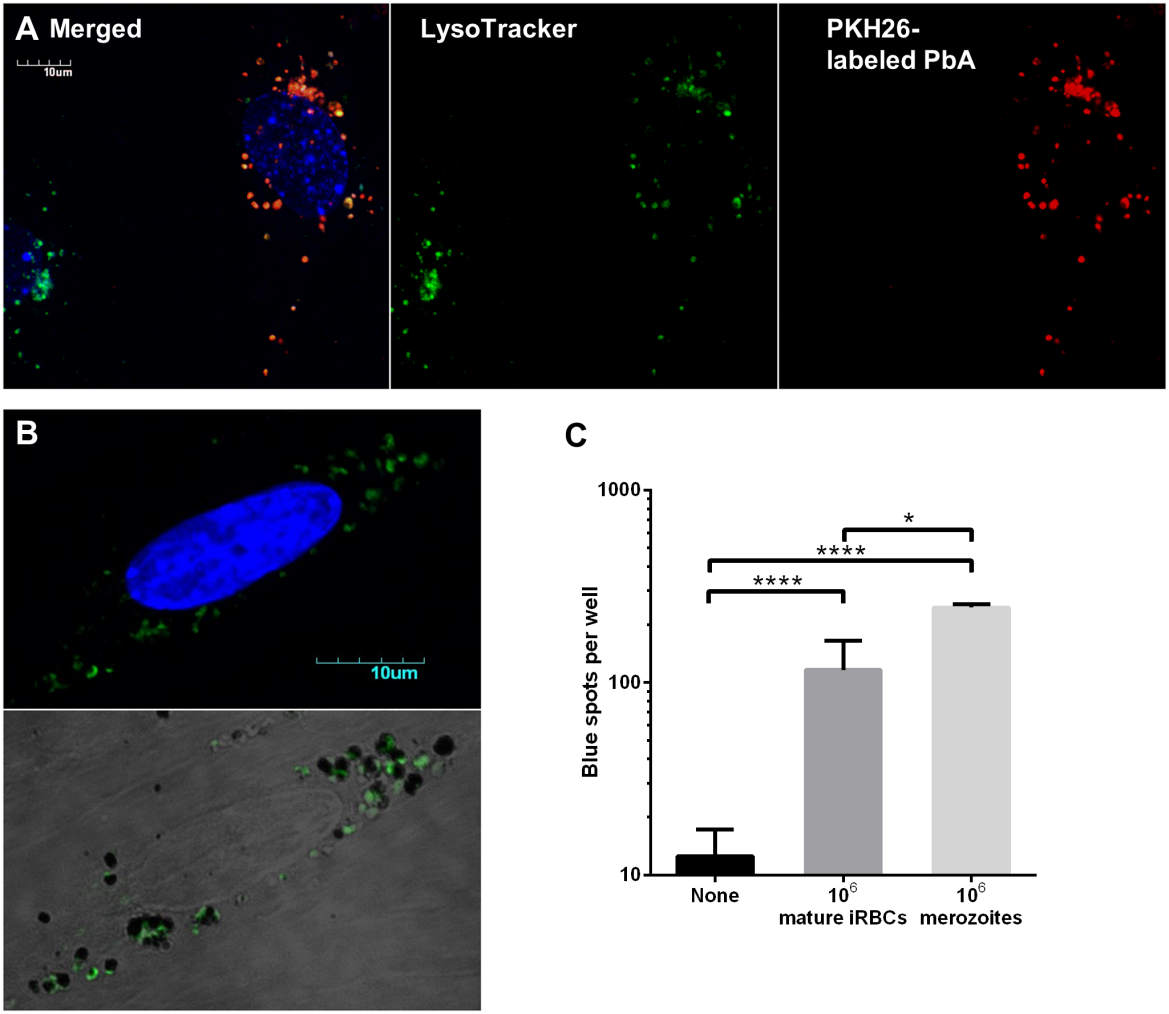
Merozoites are preferentially phagocytosed and cross-presented by MBECs. (A) Confocal microscopy of IFNγ-stimulated MBECs after overnight incubation with PKH26-labeled needle-sheared PbA mature stages (not frozen) and staining with LysoTracker Green DND-26 and Hoechst 33342. Fluorescence of extracellular PbA was quenched with trypan blue. (B) IFNγ-stimulated MBECs were incubated overnight with thawed PbA mature iRBCs, washed, fixed, permeabilized, stained with rabbit antisera raised against merozoite surface protein 1 followed by AlexaFluor488-conjugated secondary antibody and counterstained with DAPI. Confocal images were acquired using controls stained with rabbit pre-bleed sera to set the threshold. The bottom panel is an overlay of DIC and green fluorescence images adjusted for contrast, showing that green merozoites and hemozoin-containing digestive vacuoles do not co-localize. (C) No antigen, 10^6^ PbA mature iRBCs or 10^6^ PbA merozoites were added to IFNγ-stimulated MBECs. Cross-presentation was detected using LR-BSL8.4a cells 24 h later. The mature stages were freshly isolated by Percoll gradient centrifugation. A large portion was passed through a 1.2 μm filter to release the merozoites, and digestive vacuoles were depleted by MACS. *n* = 4, *P<0.05, ****P<0.0001, ANOVA with Bonferroni’s post test on log-transformed data.

To seek more evidence of phagocytosis, we labeled needle-sheared PbA mature stages (a mixture of free merozoites, late trophozoites and less mature schizonts) with PKH26, a red fluorescent membrane dye. After MBECs were incubated with PKH26-labeled parasites for 24 h, they were washed and stained with LysoTracker Green DND-26 to identify the acidic compartments (endosomes, lysosomes and phagosomes that have fused with these). The red fluorescence of parasite material that had adhered to MBECs but that had not been internalized was quenched with trypan blue [29]. Uptake of parasite material was quite heterogeneous, with some MBECs containing many red particles and others none. More importantly, red fluorescence was very well colocalized with LysoTracker (Fig 6A), indicating that the parasite material had entered the phagolysosomal pathway. To our surprise, the vesicles with colocalized red and green fluorescence were generally only 1–2 μm in diameter, consistent with the size of merozoites. Although iRBCs comprised most of the PbA material added to the wells, we did not observe any phagosomes that were large enough to contain intact mature stages (≥5 μm). To confirm that the phagosomes contained merozoites, MBECs that had been co-incubated with thawed PbA mature iRBCs were fixed and stained with rabbit antiserum recognizing merozoite surface protein 1 (MSP-1). Many of the vesicles were indeed positive for MSP-1, although under light microscopy, other dark vesicles containing malaria pigment were also observed, indicating that both merozoites and digestive vacuoles had been phagocytosed (Fig 6B).

Although the descriptive evidence suggests that MBECs phagocytosed merozoites and digestive vacuoles from ruptured schizonts, an alternative explanation could be that whole schizonts were phagocytosed, and subsequently broken down into individual merozoites and digestive vacuoles that split off into separate phagosomes. To investigate further, we compared how efficiently MBECs cross-presented an equal number of PbA mature iRBCs versus individual merozoites. One million merozoites, produced by passing mature stages through a 1.2 μm syringe filter followed by magnetic depletion of hemozoin [30], led to twice as many blue spots as one million mature iRBCs, even though the latter likely contained more than a million nascent merozoites (Fig 6C). These results support the hypothesis that merozoites are the parasite form avidly phagocytosed by MBECs and the primary source of antigen for cross-presentation. We suggest that when schizonts are added, cross-presentation efficiency is reduced by the additional requirements of rupture of the parasitophorous vacuole membrane (by parasite proteases, freeze-thaw or degradation) and merozoite egress.

### MBEC cross-presentation efficiency depends on parasite strain

**Fig 7:**
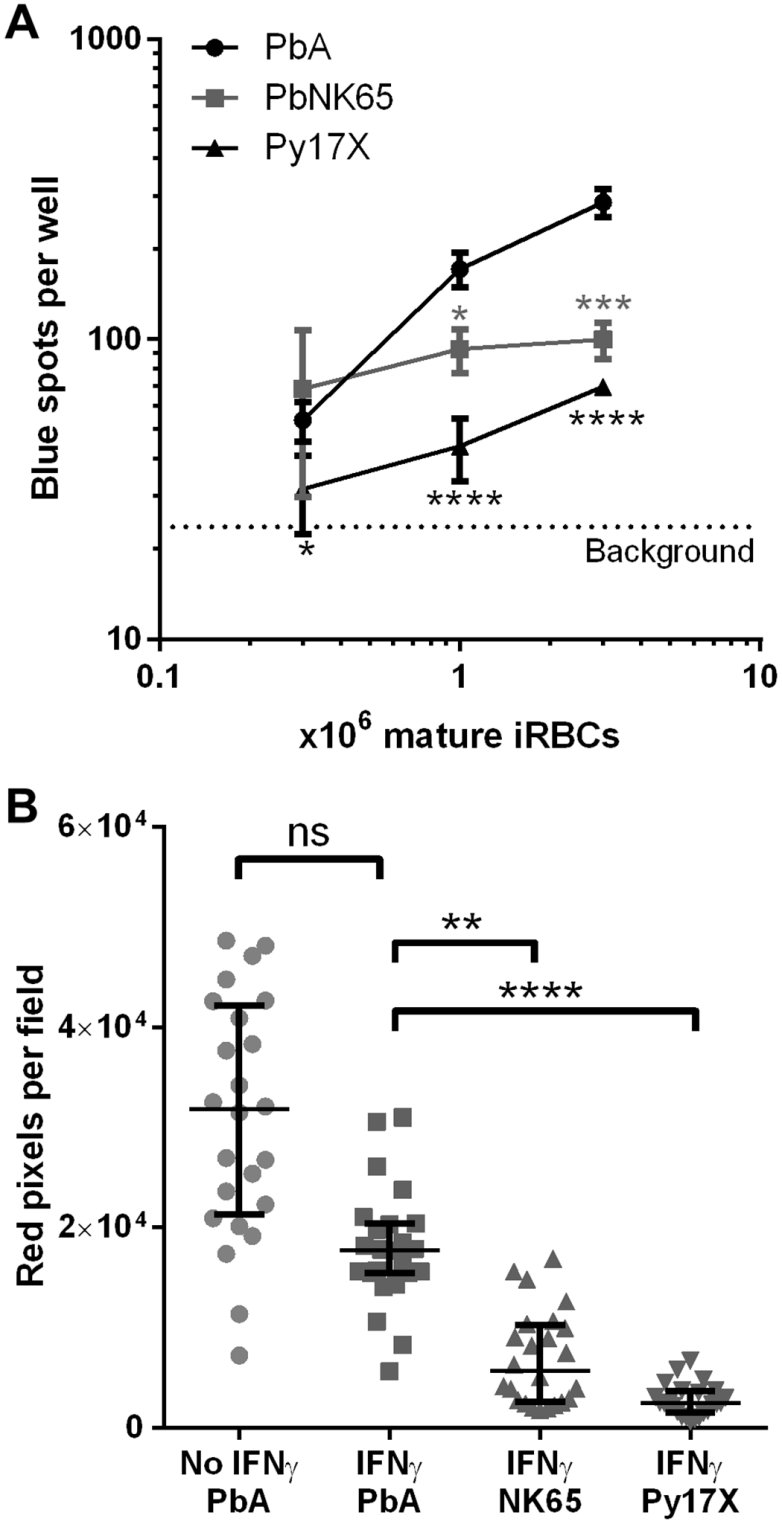
Uptake and cross-presentation efficiency of different rodent malaria parasites by MBECs *in vitro*. Mature parasite stages were isolated from the blood of mice infected with PbA, PbNK65 or Py17X by Percoll gradient centrifugation. (A) Various numbers of frozen-thawed parasites were added to IFNγ-stimulated MBECs in triplicate wells. After 24 h, the wells were washed and Pb1 cross-presentation was assayed using LR-BSL8.4a reporter cells. *P<0.05, ***P<0.001, ****P<0.0001, 2-way ANOVA with Bonferroni’s post test, comparing each of the non-ECM strains against PbA at the same dose. The dotted line indicates the background spot count from MBECs without parasites. (B) Equal numbers of frozen-thawed parasites from the three strains were labeled with PKH26 and added to unstimulated or IFNγ-stimulated MBECs in quadruplicate wells. After overnight incubation, the wells were washed and imaged by confocal microscopy (6 fields per well with 40× objective) in the presence of trypan blue to quench extracellular PKH26. The number of red pixels per field that exceed a defined brightness threshold was quantified with ImageJ. Bars represent medians and interquartile ranges. ns not significant, **P<0.01, ****P<0.0001, Kruskal Wallis test with Dunn’s post test comparing against the IFNγ + PbA group.

We have previously postulated that differences in parasite sequestration levels in the brain may explain why PbA is cross-presented by brain microvessels during infection while the non-ECM-causing parasites PbNK65 and Py17X are not [19,21]. Here, we asked if there were also intrinsic differences in how efficiently MBECs capture and cross-present iRBCs from the different parasites. The Pb1 epitope and surrounding amino acids are fully conserved in the GAP50 proteins of these three parasites, allowing this comparison to be made [19]. Percoll-isolated mature stages of the three parasites were carefully counted and added at three different doses to IFNγ-stimulated MBECs. Significantly less cross-presentation was detected from Py17X compared to PbA at all doses (Fig 7A). PbNK65 was cross-presented with intermediate efficiency but had a relatively flat dose response curve, such that the cross-presentation readout was about one-third that of PbA at the highest dose. We infer that differences in both per-iRBC cross-presentation efficiency and local parasite abundance in the brain may combine such that only PbA induces a pathogenic, detectable level of MBEC cross-presentation during infection. To further investigate whether the differences in cross-presentation efficiency arose from differential uptake of the parasite strains, equal numbers of PKH-labeled PbA, NK65 or Py17X mature stages were added to MBECs. After overnight incubation, the wells were washed and imaged in the presence of trypan blue to quench extracellular and quantify intracellular PKH26 fluorescence. The amounts of internalized NK65 and Py17X were significantly lower than that of PbA (Fig 7B), and the relative levels suggest that this may be the primary factor behind the reduced cross-presentation efficiencies. The uptake level of PbA in MBECs that had not been stimulated with IFNγ was also quantified. Unexpectedly, IFNγ stimulation did not increase parasite uptake and may even have decreased it slightly, although this did not reach statistical significance (Fig 7B).

### Human brain endothelial cells phagocytose merozoites

**Fig 8:**
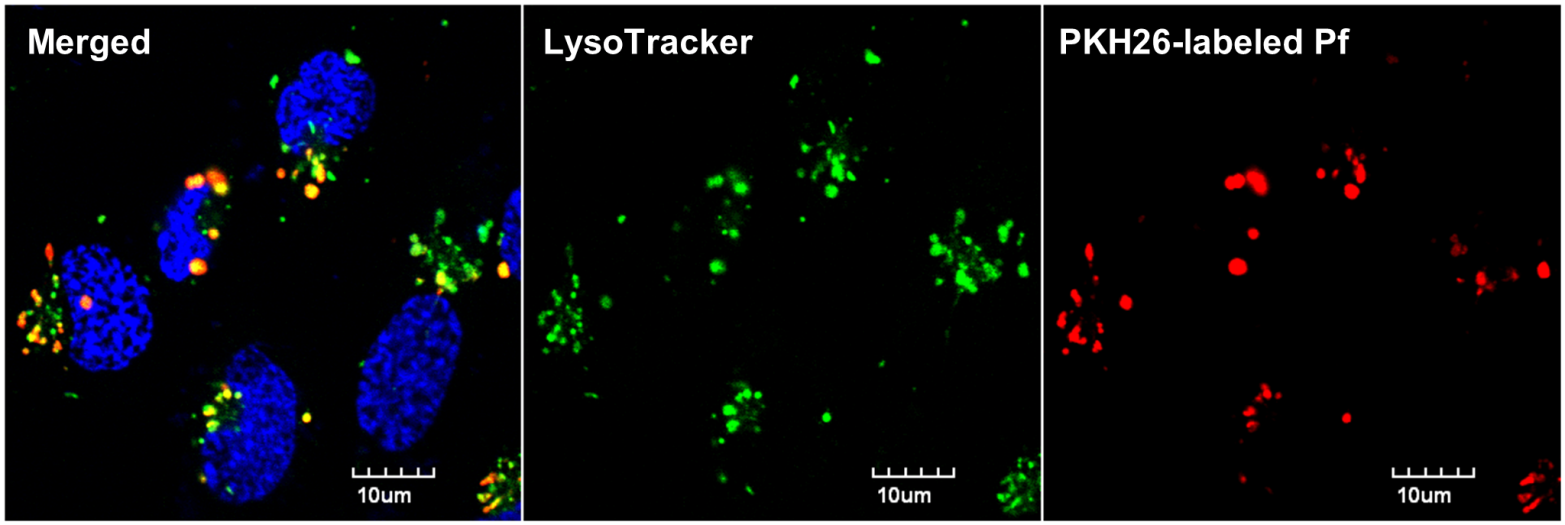
Human brain endothelial cells phagocytose Pf merozoites *in vitro*. PKH26-labeled Pf merozoites were incubated with IFNγ-stimulated hCMEC/D3 cells for 24 h. The cells were then washed thoroughly and stained with Lysotracker Green DND-26 and Hoechst 33342 prior to confocal imaging. Extracellular PKH26 fluorescence was quenched with trypan blue.

Currently, the lack of known blood-stage Pf epitopes and cognate TCRs is a barrier to determining whether human brain endothelial cells cross-present parasite antigen. Instead, we asked if human brain endothelial cells can at least phagocytose merozoites. The hCMEC/D3 line was stimulated with IFNγ and co-incubated with PKH26-labeled Pf merozoites for 24 h. Confocal fluorescence imaging was performed after LysoTracker and Hoechst staining, with trypan blue quenching of unphagocytosed parasites. We observed that almost all the hCMEC/D3 nuclei (82/89) were associated with red fluorescent vesicles 1–2 μm in diameter that colocalized with LysoTracker (Fig 8). We conclude that merozoites were avidly phagocytosed by hCMEC/D3 cells, leaving open the possibility that brain endothelial cross-presentation of parasite antigen may play a role in the pathogenesis of human cerebral malaria.

## Discussion

More than a decade ago, we and others quantified the migration of CD8^+^ T cells to the brain during ECM and speculated that they may exert their cytotoxic effector functions on antigen-presenting endothelial cells [5,6]. Although the ability to acquire and present exogenous antigens on MHC class I molecules was originally thought to be limited to professional antigen-presenting cells, it has become increasingly clear that endothelial cells are conditional antigen-presenting cells that cross-present when activated by danger signals or cytokines (reviewed by Mai et al. [31]). Nevertheless, evidence of endothelial presentation of malarial antigens has been elusive, in part because of the lack of known MHC class I epitopes and methods to detect them. Some circumstantial evidence was provided by Jambou *et al*. demonstrating that a human brain endothelial cell line could acquire proteins from Pf iRBCs [32]. We recently developed reporter cells for detecting three immunogenic class I epitopes from PbA and found that microvessel fragments from the brains of ECM-afflicted mice presented them [19,21]. However, the question of whether endothelial cells were the cross-presenting cell type remained unresolved.

Here, we present definitive evidence that brain endothelial cells in PbA-infected mice cross-present PbA antigens during ECM. Whole mouse brains were dissociated using papain and sorted into different cell populations, of which only endothelial cells and CD45^+^ cells presented Pb1 peptide. We had also previously considered pericytes to be candidate cross-presenting cells. Pericytes are phagocytic conditional antigen-presenting cells [33] and are embedded within the basal lamina, in line with the importance of collagenase treatment for detecting cross-presentation [19]. In fact, we found that murine brain pericytes cultured *in vitro* had the capacity to cross-present Pb1 peptide from PbA iRBCs when stimulated with IFNγ (S4 Fig). However, we did not detect cross-presentation from pericytes isolated from PbA-infected mice, suggesting that pericytes had limited access to iRBCs *in vivo*. We cannot exclude the possibility that a low level of pericyte cross-presentation, below the assay detection limit, occurred at endothelium breach sites. Amongst the CD45^+^ population, peripheral leukocytes and/or PVM but not microglial cells were found to cross-present PbA antigen, pointing again to the importance of antigen accessibility. We have previously shown that total brain-sequestered leukocytes cross-presented an order of magnitude less than brain microvessel fragments [19]. Here, the similar numbers of blue spots elicited by sorted endothelial cells and leukocytes can be attributed to the low yield of unicellular endothelial cells, and perhaps reduced cross-presentation ability after disruption of cell-cell junctions. Normalizing the spot counts by the number of sorted cells added makes it clear that MBECs cross-present PbA antigen during ECM and are the main cell type responsible for the results of the brain microvessel cross-presentation experiments. Cross-presentation by brain-sequestered leukocytes is unlikely to play a major role during the effector phase of ECM, as mice depleted of macrophages and dendritic cells from day 5 onwards using the MAFIA (macrophage Fas-induced apopotosis) transgenic mouse system still succumb to ECM and exhibit undiminished brain microvessel cross-presentation [19].

Because of the crucial role of endothelial cells in forming the blood-brain barrier, which is disrupted during ECM, and evidence of their apoptosis in a perforin-dependent manner [34], endothelial cell killing has been proposed to be major pathogenic process in ECM. Endothelial cell apoptosis during ECM has since been confirmed by other approaches [35,36] but has also been disputed [37]. We show here that MBECs cross-presenting PbA antigen *in vitro* are killed by CD8^+^ T cells induced during infection. However, while this experiment demonstrates the capacity of such cytolysis to occur, the high CD8^+^ T cell to endothelial cell ratio used *in vitro* is not intended to reflect the small number of CD8^+^ T cells found in the brain *in vivo* [5]. Direct killing of endothelial cells may not be the only (or even major) mechanism of blood-brain barrier disruption by CD8^+^ T cells. While CD8^+^ T cells were previously thought to be present only within the vasculature [38], intravital microscopy has recently revealed that some CD8^+^ T cells extravasate during ECM [39]. Our olfactory bulb smears confirm that CD8^+^ T cells are found in contact with both luminal and abluminal faces of endothelial cells as well as in the parenchyma. This suggests an alternative explanation for the importance of perforin and Granzyme B during ECM. Granzyme B plays an important role in breaking down the basement membrane during diapedesis by cytotoxic lymphocytes [40]. Transgenic CD8^+^ T cells have been shown to cross the blood-brain barrier at sites where the cerebral endothelium presented their cognate epitope [41]. Perforin-expressing CD8^+^ T cells have also been shown to disrupt tight junctions between endothelial cells pulsed with their cognate peptide [42,43]. Therefore, we propose that endothelial cells cross-presenting PbA antigen *in vivo* may promote CD8^+^ T cell extravasation and thus remodeling of the basement membrane and tight junctions in a perforin- and Granzyme B-dependent manner. The relative importance of this proposed mechanism versus endothelial cell apoptosis during ECM remains unclear.

We have shown here that MBEC cross-presentation of malarial antigen requires IFNγ both *in vivo* and *in vitro*. IFNγ can be viewed as the central cytokine coordinating the key players in ECM pathogenesis, directing CD8^+^ T cell migration to the brain [15,44], mediating parasite sequestration [45,46], and now enabling endothelial cross-presentation of PbA antigen. These three phenomena develop coincidentally in the brain 6 days post-infection [19,46], just prior to and probably precipitating the development of neurological signs. Note that because parasite biomass in the brain is significantly reduced in IFNγ-deficient mice [45,46], it is not clear whether the lack of brain microvessel cross-presentation in these mice results from low antigen availability or low endothelial cross-presentation ability or both. Our *in vitro* experiments conducted in the presence of ample parasite material are consistent with IFNγ stimulation causing endothelial cells to gain the ability to cross-present phagocytosed material. The role of IFNγ in enhancing antigen processing and presentation has been well studied. In the context of class I antigen presentation, IFNγ has been shown to upregulate immunoproteasome subunits, proteasome activator 28, TAP, tapasin, MHC class I heavy chains, β_2_-microglobulin (reviewed previously [47]) and ERAP1 [38]. Microarray analysis has revealed that genes involved in antigen processing and presentation, including TAP and β_2_-microglobulin, are upregulated in the brains of mice with ECM compared to infected ECM-resistant BALB/c mice [48]. In addition, we speculate that IFNγ may play a role in enhancing endothelial uptake of malarial antigens *in vivo* by upregulating receptors associated with adhesion and/or phagocytosis. Parasite cytoadherence to brain endothelial ICAM-1 via Pf erythrocyte membrane protein 1 (PfEMP1) is strongly implicated in human cerebral malaria [49]. The picture is less clear in ECM as PbA lacks PfEMP1 orthologues, and a recent *in vitro* model of PbA adhesion to immortalized MBECs was unaffected by ICAM-1 blocking [50]. With primary MBEC cultures, we observed that although IFNγ stimulation did not enhance parasite phagocytosis under static conditions, it increased surface expression of ICAM-1 (S3C Fig). ICAM-1 also mediates leukocyte adhesion, and in particular, is a ligand for LFA-1 highly expressed by PbA-specific CD8^+^ T cells in the brain [21].

Unlike IFNγ, LTα was not required for brain microvessel cross-presentation during infection. While LTα-deficient mice have been shown to be ECM-resistant [23], the role of LTα in ECM remains poorly understood. In wild-type mice, we expect some of the cross-presenting brain endothelial cells to be killed by cognate CD8^+^ T cells, thus reducing the readout of the brain microvessel cross-presentation assay. Thus, both ECM resistance and the increased brain microvessel cross-presentation signal in LTα-deficient mice could be explained by a deficiency in the number or cytotoxicity of antigen-specific CD8^+^ T cells in the brain. This remains to be investigated, but since LTα-deficient mice lack peripheral lymph nodes and have spleens with disrupted microarchitecture and severely reduced numbers of dendritic cells [51,52], induction of the T cell response is likely to be affected.

By studying primary cultures of MBECs, we have determined that endothelial cross-presentation of PbA antigen occurs by the phagosome-to-cytosol route and requires both TAP and proteasomes. This is not surprising as aortic endothelial cells have been reported to cross-present an unrelated antigen by the same mechanism [53]. Elucidation of the cross-presentation mechanism may suggest targets for adjuvant therapy of cerebral malaria by reducing the vulnerability of brain endothelial cells to CD8^+^ T cell-mediated cytolysis. Since the *in vitro* experiments were largely performed with freeze-thawed iRBCs, and no cross-presentation was observed with a Transwell format, we can formally conclude that the antigen presentation was true cross-presentation: the MBECs were not invaded by parasites, nor were the peptide epitopes already processed in the parasite rather than the MBECs. As both platelets [54] and microparticles [55,56] have been reported to contain parasite-derived proteins during malaria infection, we asked if either could be alternative sources of antigen cross-presented by brain endothelial cells. Neither platelets nor microparticles purified from infected mouse blood induced MBECs to cross-present Pb1 *in vitro* (S3C Fig); however, we cannot rule out that platelets and microparticles may contain other antigens that can be cross-presented.

Unexpectedly, our results suggest that MBECs phagocytosed free merozoites and digestive vacuoles rather than whole iRBCs *in vitro*. Merozoites and digestive vacuoles may be released from schizonts by membrane rupture after freeze-thaw, or in the case of freshly isolated mature stages, by continued schizont maturation and rupture or degradation *in vitro*. Purified merozoites were more efficient at inducing cross-presentation of Pb1 than the same number of mature iRBCs (even though each mature schizont contains 12–18 merozoites), implying that free merozoites are likely to be the major source of cross-presented antigen. While Pb1 originates from a merozoite-expressed protein (GAP50), it is likely that digestive vacuoles also contribute other antigens, e.g. Pb2-containing bergheilysin [21], for cross-presentation as they are avidly phagocytosed. It remains to be seen whether merozoites rather than iRBCs are favored for phagocytosis by endothelial cells *in vivo*, in mice and humans. While we have not observed any unambiguous instances of MBECs phagocytosing whole PbA iRBCs *in vitro*, it has been reported that Pf iRBCs first transfer membrane material to and are then engulfed by a human brain endothelial cell line [32]. In our hands, the same cell line avidly phagocytosed Pf merozoites, suggesting that our findings with PbA may have relevance to human disease. Our observation that PbNK65 and Py17X iRBCs were less efficiently internalized and cross-presented than PbA suggests that merozoite phagocytosis by MBECs may be at least partially ligand-specific. It is unlikely that opsonins were responsible for differential phagocytosis of the parasite strains as all three were washed and cultured overnight in medium lacking mouse serum. Much effort has been dedicated to studying the interactions between schizonts and endothelial cells, but we propose that research into merozoite-endothelial interactions, particularly defining any endothelial receptors mediating phagocytosis, could open up new avenues for adjunct therapy of cerebral malaria.

## Materials and Methods

### Ethics statement

All animal experiments were approved by the Institutional Animal Care and Use Committee (IACUC #110630) and complied with the guidelines of the Agri-Food and Veterinary Authority (AVA) and the National Advisory Committee for Laboratory Animal Research (NACLAR).

### Mice

C57BL/6J mice (6–8 weeks old) were used for infection experiments, sex-matched to knockout mice in the same experiment. C57BL/6J mice up to 12 weeks old were used for MBEC culture. C57BL/6J mice deficient for IFNγ, TNFα, LTα and TAP1 originated from the Jackson Laboratory. All mice were bred and housed under specific pathogen-free conditions in the Biomedical Resource Centre, Singapore.

### Rodent parasites, infection and *in vitro* maturation

Mice were infected with *P*. *berghei* ANKA clone 15Cy1 (PbA) by intraperitoneal injection of 0.5–1 × 10^6^ iRBCs. Details of this and other rodent strains were previously published [19]. To obtain mature stages, infected blood was diluted in PBS, passed through a Plasmodipur filter (EuroProxima) to remove leukocytes, centrifuged and resuspended at 2–4% hematocrit in RPMI medium containing 20% FCS and penicillin/streptomycin (P/S). The blood was cultured overnight at 37°C on a rocking platform in non-vented flasks flushed with gas mixture (5% CO2, 5% O2, 90% N2). Schizonts and late trophozoites were isolated from the interface formed by gradient centrifugation (1450 × g, 10 min) over 65% Percoll, washed, aliquoted and frozen in parasite medium at -80°C.

### Reporter cells

For most experiments, the cloned TCR-transduced cell line LR-BSL8.4a was used to detect presentation of the Pb1 epitope SQLLNAKYL in the context of H-2D^b^, with TCR ligation inducing *lacZ* expression [19]. We recently generated two other reporter cell lines, LR-BSL13.6b and LR-WH3.4, which recognize the Pb2 and F4 epitopes [21]. All reporter cells were cultured in RPMI complete medium supplemented with 10% FBS, 1 mM sodium pyruvate, 50 μM β-mercaptoethanol, P/S and Primocin (Invivogen).

### *Ex vivo* brain microvessel cross-presentation assay

We described this protocol in detail previously [19]. In brief, brain microvessel fragments were isolated from terminally exsanguinated mice by dextran gradient centrifugation and capture on a cell strainer. These fragments were digested with collagenase prior to overnight co-incubation with LR-BSL8.4a reporter cells. Cross-presentation was measured by counting blue spots following X-gal staining.

### Antibodies

Antibodies against mouse CD45 (clone 30-F11, FITC), CD31 (clone 390, PE-Cy7) and CD11b (clone M1/70, PerCP-Cy5.5) were purchased from BD Biosciences, Biolegend, and eBioscience respectively and were used at 1 μg/ml. αCD140b-APC (Miltenyi) was used at 1:10 dilution. Rabbit polyclonal antibodies against NG2 chondroitin sulfate proteoglycan and von Willibrand Factor were purchased from Millipore and used at 2–5 μg/ml for flow cytometry and immunocytofluorescence. Unconjugated rat antibodies against CD8α (clone YTS169.4) and CD8β (clone 53–5.8) from Bio X Cell were used at 5 μg/ml for immunofluorescence. *P*. *yoelii* PyMSP-1/19 Rabbit Antiserum (cross-reactive with PbA MSP-1), MRA-23, deposited by JH Adams (University of South Florida, FL, USA), was obtained through the MR4 as part of the BEI Resources Repository, NIAID, NIH (Bethesda, MD, USA). Goat anti-rabbit secondary antibodies conjugated with Alexa Fluor 488 and Alexa Fluor 647 and goat anti-rat antibody conjugated with Alexa Fluor 555 were from Life Technologies and were used at 1:1000–1:5000 dilution.

### Identification of cross-presenting cell types in the brain

A papain-based Neural Tissue Dissociation Kit (Miltenyi) was used in conjunction with a gentleMACS Octo Dissociator with Heater to obtain unicellular suspensions from brains of terminally exsanguinated mice. The papain volume was reduced to 15 μL per brain to reduce cleavage of MHC class I molecules and CD31. The resulting homogenate was diluted in DMEM complete medium (high glucose, with pyruvate, supplemented with 10% FBS and P/S) and passed through a 100 μm cell strainer, then centrifuged at 1900 × g for 10 min over a 30% Percoll gradient to remove myelin. The pelleted cells were washed and stained for CD45, CD31 and CD140b in 50 μL, and then red blood cells were lysed by dilution with 200 μL of ACK buffer. Washed cells were then sorted on a FACSAria II (BD Biosciences), after gating on DAPI-excluding singlet cells, into 4 populations: CD45^+^, CD45^-^CD31^+^CD140b^-^, CD45^-^CD31^-^CD140b^+^ and triple-negative. To isolate microglia and peripheral leukocytes from brains, papain was avoided and each brain was manually mashed and digested in 10 mL PBS with 5 mg collagenase 4 and 0.1 mg DNaseI for 30 min at room temperature. Myelin removal and staining were performed similarly, except that CD45 and CD11b antibodies were used to sort CD45^int^CD11b^+^ microglia and all CD45^hi^ leukocytes. All the cells of each gated population per brain were pelleted, resuspended in 100 μL RPMI complete medium and seeded in one well of a 96-well round bottom plate. LR-BSL8.4a cells (3 × 10^4^ cells in 100 μL medium) were added to each well and the plate was incubated overnight (for collagenase-digested cells) or 24 h (for papain-digested cells) before the cells were transferred to a 96-well filter plate for X-gal staining.

### MBEC primary culture

Brain microvessels were isolated from naïve C57BL/6J mice as for the *ex vivo* cross-presentation assay, but collagenase/DNaseI digestion was lengthened to 3 h at 37°C. Digested vessels were triturated with a 10 mL serological pipette and washed twice with DMEM complete medium. Culture conditions were adapted from Lu et al. [57]. The vessel fragments were resuspended in endothelial medium (DMEM complete with added MEM non-essential amino acids, 0.1 mg/ml heparin and 0.1 mg/ml endothelial cell growth supplement from Corning) with 4 μg/ml puromycin and seeded in a collagen-coated 48-well plate. Typically, 5 brains were used to seed 32 wells. The medium was replaced (without puromycin) in the morning 3 days later and every 3–4 days thereafter.

### MBEC cross-presentation *in vitro*

MBECs were stimulated with 10 ng/ml recombinant mouse IFNγ (R&D Systems) 24 h before parasites (3 × 10^6^ freeze-thawed PbA mature iRBCs unless otherwise stated) were added. After a further 24 h, the MBECs were washed and 6 × 10^4^ reporter cells in 0.4 ml RPMI complete medium were added. Following overnight co-incubation, the reporter cells were resuspended and moved to a 96-well filter plate for X-gal staining.

### Fluorescence imaging of MBECs

For immunofluorescence and live fluorescence imaging, MBECs were cultured in 8-well Ibidi tissue culture μ-slides that we coated with collagen I. For immunofluorescence assays, cells were fixed with 2% formaldehyde in PBS for 20 min, permeabilized with 0.1–0.5% Triton X-100 in PBS for 10 min, and blocked with 10% goat serum for 30–60 min prior to staining. The buffer used for washing and staining was PBS with 0.5% BSA, and 0.05% Tween 20 was also added to reduce non-specific staining with MSP-1 antiserum. Slides were stained overnight at 4°C with the primary antibody, stained for 1 h at room temperature with the secondary antibody, counterstained with DAPI and mounted in FluorSave (EMD Millipore). For live fluorescence imaging, freshly isolated PbA mature iRBCs were repeatedly forced through a 29G needle to yield a mixture of late trophozoites, early schizonts and merozoites. The parasite material was labeled with 2 μM PKH26 (Sigma-Aldrich) according to the manufacturer’s protocol, and added to IFNγ-stimulated MBECs for 24 h. The cells were then washed thrice before labeling with 50 nM LysoTracker Green DND-26 (Life Technologies) and 8 μM Hoechst 33342 for 5 min at 37°C. After washing, the cells were covered with medium containing 0.5 mg/ml trypan blue to quench extracellular PKH26 fluorescence [29] and imaged immediately. Images were acquired on an Olympus FV1000 confocal microscope with a 100x objective lens; ImageJ was used for overlaying and cropping only unless otherwise specified.

### CD8^+^ T cell killing *in vitro*

Confluent MBECs in 9 wells of a 48-well plate were stimulated with 10 ng/ml IFNγ, and 3 × 10^6^ thawed PbA mature iRBCs were added to 6 wells 24 h later. The next day, CD8^+^ T cells were isolated by negative magnetic selection (Miltenyi) from the spleens of a naïve C57BL/6J mouse and one infected with PbA 6 days previously. The MBEC wells were washed and aspirated 24 h after parasite addition, and 10^6^ CD8^+^ T cells were added in RPMI complete medium containing 50 U/ml IL-2. The wells were gently washed and imaged (10× objective, DIC) after 20 h co-incubation.

### Immunofluorescence on olfactory bulb smears

Olfactory bulbs were dissected from terminally exsanguinated mice and each lobe was smeared between two glass slides. After air-drying, the bottom slide was fixed in acetone at -20°C for 5–10 min. Blocking, incubation with primary antibodies (against von Willebrand factor, CD8α and CD8β) and secondary antibodies (anti-rabbit Alexa Fluor 488 and anti-rat Alexa Fluor 555) was performed as described above.

### PbA merozoites

PbA mature stages (from 5 infected mice) isolated by Percoll gradient centrifugation were resuspended in 3 ml of parasite medium and passed through a 1.2 μm Acrodisc syringe filter (Pall) to release the merozoites [58]. The filtrate was passed twice through an LS magnetic column (Miltenyi) to deplete hemozoin-containing digestive vacuoles [30]. Merozoites in the flowthrough were concentrated by centrifugation at 4000 × g for 10 min, after which a small aliquot was stained with 8 μM Hoechst dye and 5 μg/ml dihydroethidium for quantification on a MACSQuant Analyzer (Miltenyi).

### Human brain endothelial cells and Pf merozoites

The immortalized human brain endothelial cell line hCMEC/D3 [59], a kind gift of Pierre-Olivier Couraud (Institut Cochin, Paris, France), was cultured in EGM-2 MV medium (Lonza) on collagen-coated plastic. Pf (clone 3D7) was cultured in RPMI-Albumax II as previously described [60]. A schizont stage 3D7 culture was enriched with an LD magnetic column (Miltenyi), treated with 10 μm epoxysuccinyl-L-leucylamido(4-guanidino) butane (E-64) for 6 h, then passed through a 1.2 μm Acrodisc syringe filter [58]. The released merozoites were labeled with 2 μm PKH26 and added to hCMEC/D3 cells that had been stimulated with 50 ng/ml recombinant human IFNγ (R&D Systems) for 20 h prior. After 24 h, the cells were washed, stained with LysoTracker and Hoechst, and imaged as above for MBECs.

### Statistical analysis

Blue spots counts were analyzed by unpaired t-test (2 groups), 1-way ANOVA (3 or more groups with one independent variable) or 2-way ANOVA (2 independent variables) after logarithmic transformation to achieve homoscedasticity and normal distribution. Bonferroni’s post test was used to compare groups following ANOVA. The exceptions were the cross-presentation experiments using sorted cell populations (Mann-Whitney U test) and quantification of parasite uptake (Kruskal-Wallis test) where non-parametric tests were employed as the data were not normally distributed. Error bars represent standard deviations unless otherwise stated.

## Supporting Information

S1 Fig Representative FACS plots for sorting brain cell types. (A) FACS data from a PbA-infected mouse brain processed with the Neural Tissue Dissociation Kit. Live singlet cells were divided into CD45^-^ and CD45^+^ populations (left). The CD45^-^ cells were further gated into CD31^+^ endothelial cells, CD140b^+^ pericytes and triple-negative cells (right). (B) Naïve (left) or PbA-infected (right) mouse brains were mashed and digested with collagenase prior to sorting for CD45^int^CD11b^+^ microglial cells and CD45^hi^ leukocytes.
(TIF)

S2 Fig MBEC cross-presentation and effects of IFNγ stimulation. (A–B) Cross-presentation of other PbA epitopes. MBECs were stimulated (or not) with 10 ng/ml IFNγ 24 h prior to addition of 3 × 10^6^ PbA mature iRBCs. After another 24 h, the wells were washed and one of the following reporter cell lines (6 × 10^4^ cells) were added: (A) LR-BSL13.6b, recognizing the Pb2 epitope IITDFENL, (B) LR-BSLWH3.4, recognizing the F4 epitope EIYIFTNI. X-gal staining was performed after overnight co-incubation. *n* = 3, **P<0.01, ANOVA with Bonferroni’s post test on log-transformed data. (C) Cytokines (10 ng/ml each) were added to MBECs 24 h before 10^6^ PbA mature iRBCs were added. 24 h later, LR-BSL8.4a reporter cells were used to detect Pb1 cross-presentation. The “No PbA ctrl” well was stimulated with IFNγ. *n* = 3, ****P<0.0001, ANOVA with Bonferroni’s post test on log-transformed data. (D) MBECs were stimulated (or not) with 10 ng/ml IFNγ 24 h prior to addition of 0.3 mg/ml ovalbumin. Wells were washed and assayed for cross-presentation 24 h later using reporter cells expressing the OT-I TCR. As the spot numbers after X-gal staining were too numerous to count, the well images were analyzed with the color threshold function of ImageJ to quantify the number of blue pixels. *n* = 3, ***P<0.001, ****P<0.0001, ANOVA with Bonferroni’s post test. (E) MBECs were analyzed by flow cytometry 24 h after incubation with or without 10 ng/ml IFNγ. Histograms for unstained cells (grey fill), unstimulated cells (dotted line) and IFNγ-stimulated cells (red line) are overlaid. (F) To test whether proteasome inhibitors affected MHC class I expression, IFNγ-stimulated MBECs were incubated with no inhibitor, 10 μM lactacystin, 10 μM MG132 or 100 nM bortezomib for 6 h in the presence of 10^−5^ ug/ml Pb1 peptide. The wells were then washed and assayed for Pb1 presentation using LR-BSL8.4a cells. As the spots were too numerous to count, the number of blue pixels was quantified as for (D). *n* = 3, no significant difference by ANOVA.
(TIF)

S3 Fig Sources of cross-presented antigen that were ruled out. (A) IFNγ-stimulated MBECs were incubated with nothing, 3 × 10^6^ uninfected RBCs (uRBCs) from a naïve mouse or 3×10^6^ PbA mature iRBCs for 24 h, after which cross-presentation of the Pb1 epitope was assayed using LR-BSL8.4a reporter cells. *n* = 4, ns not significant, ****P<0.0001, ANOVA with Bonferroni’s post test on log-transformed data. (B) MBECs were grown in 24-well plates and stimulated with IFNγ. 6×10^6^ PbA mature iRBCs were added directly to one group of wells. Transwell inserts (0.4 μm pore size) were added to another group of wells, after which the same number of iRBCs were added to the upper chamber. Cross-presentation of Pb1 epitope was assayed after 24 h. *n* = 3, ***P<0.001, ****P<0.0001, ANOVA with Bonferroni’s post test on log-transformed data. (C) Blood from PbA-infected mice was collected in sodium citrate and sequentially centrifuged at 1500 × g for 15 min (removing RBCs and merozoites), twice at 17 000 × g for 4 min (pelleting the platelets) and at 17 000 × g for 1 h (pelleting the microparticles). Platelets and microparticles coming from 200 μl of plasma were added to wells of IFNγ-stimulated MBECs for 24, after which cross-presentation of Pb1 was measured. *n* = 4, no significant difference by ANOVA on log-transformed data.
(TIF)

S4 Fig Pericytes cross-present PbA antigen in vitro after IFNγ stimulation. Pericytes were cultured from mouse brain microvessels in two different ways (see below). They were stimulated (or not) with 10 ng/ml IFNγ 24 h prior to addition (or not) of 3 × 10^6^ thawed PbA mature iRBCs. After 24 h, the wells were washed and 6 × 10^4^ LR-BSL8.4a cells were co-incubated overnight, then stained with X-gal. The spot counts were analyzed by ANOVA and Bonferroni’s post test after log transformation. (A) Mouse brain microvessels were cultured in endothelial medium without puromycin selection. When confluent, the cells were detached and sorted for CD45^-^CD31^-^NG2^+^ pericytes, which were seeded in a 48-well plate in complete DMEM medium. The cross-presentation assay was conducted after 2 weeks of growth. *n* = 3, **P<0.01. (B) Mouse brain microvessels were cultured in endothelial medium without puromycin, passaged once in endothelial medium and twice in Pericyte Medium (ScienCell). There were essentially no CD45^+^ or CD31^+^ cells after this. *n* = 4, ****P<0.0001.
(TIF)
