# Supplementary figures and images for: Activated Brain Endothelial Cells Cross-Present Malaria Antigen
Source: PLoS Pathog. 2015 Jun 5;11(6):e1004963. doi: 10.1371/journal.ppat.1004963 (PMC4457820; doi:10.1371/journal.ppat.1004963)

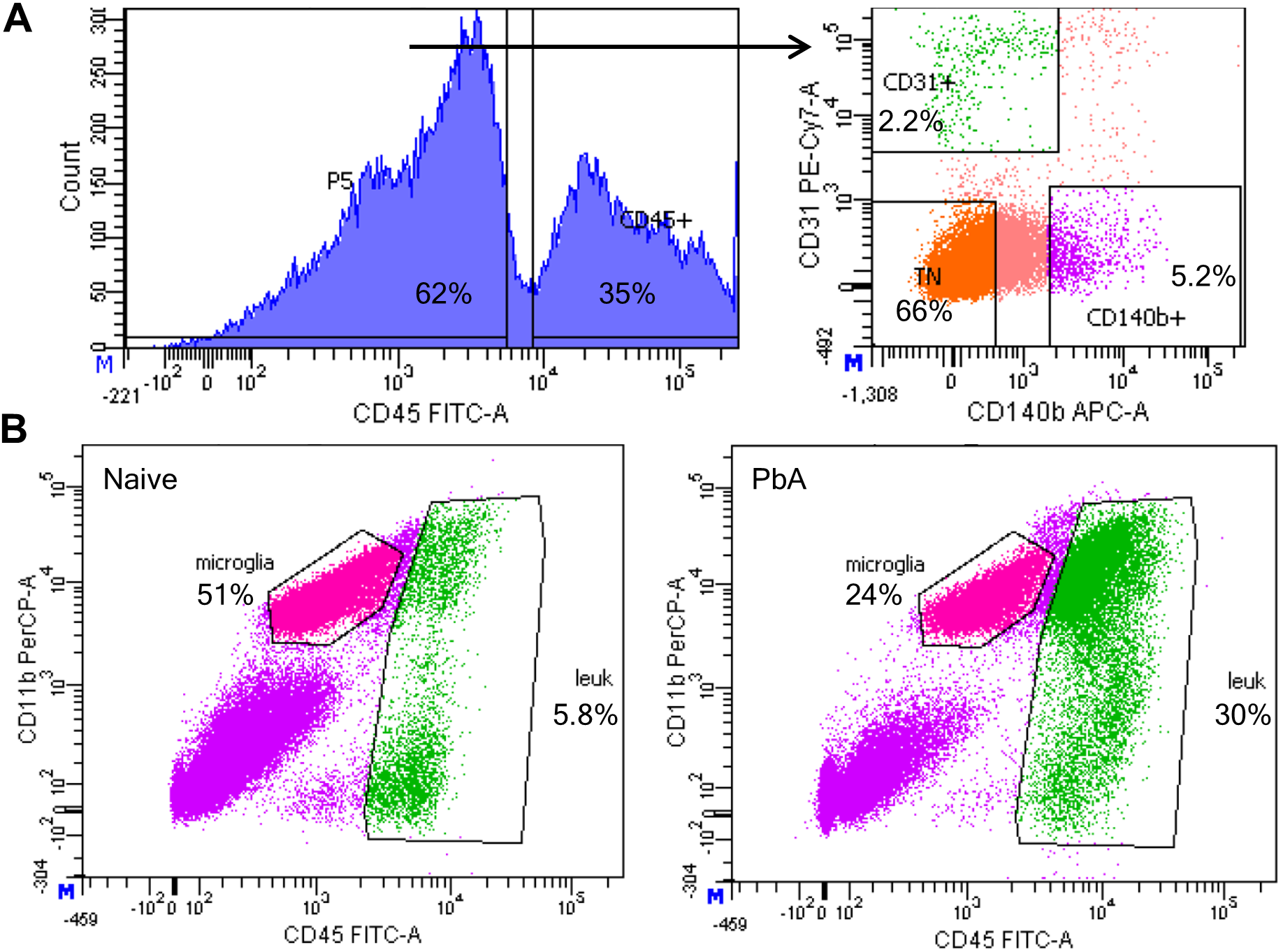

Supplement: S1 Fig — (A) FACS data from a PbA-infected mouse brain processed with the Neural Tissue Dissociation Kit. Live singlet cells were divided into CD45- and CD45+ populations (left). The CD45- cells were further gated into CD31+ endothelial cells, CD140b+ pericytes and triple-negative cells (right). (B) Naïve (left) or PbA-infected (right) mouse brains were mashed and digested with collagenase prior to sorting for CD45intCD11b+ microglial cells and CD45hi leukocytes. (TIF) [file ppat.1004963.s001.tif]

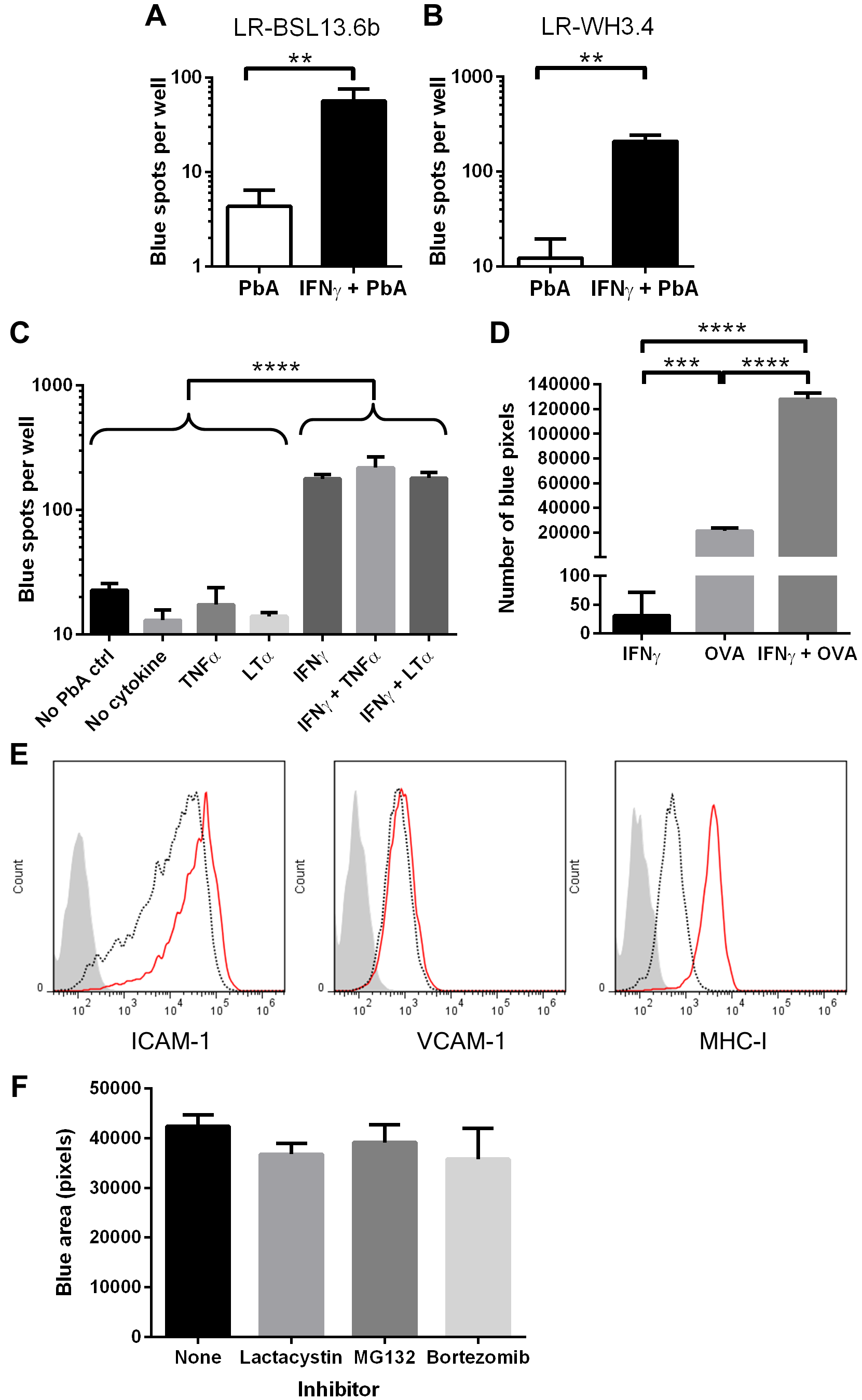

Supplement: S2 Fig — (A–B) Cross-presentation of other PbA epitopes. MBECs were stimulated (or not) with 10 ng/ml IFNγ 24 h prior to addition of 3 × 10^6^ PbA mature iRBCs. After another 24 h, the wells were washed and one of the following reporter cell lines (6 × 10^4^ cells) were added: (A) LR-BSL13.6b, recognizing the Pb2 epitope IITDFENL, (B) LR-BSLWH3.4, recognizing the F4 epitope EIYIFTNI. X-gal staining was performed after overnight co-incubation. n = 3, **P<0.01, ANOVA with Bonferroni’s post test on log-transformed data. (C) Cytokines (10 ng/ml each) were added to MBECs 24 h before 106 PbA mature iRBCs were added. 24 h later, LR-BSL8.4a reporter cells were used to detect Pb1 cross-presentation. The “No PbA ctrl” well was stimulated with IFNγ. n = 3, ****P<0.0001, ANOVA with Bonferroni’s post test on log-transformed data. (D) MBECs were stimulated (or not) with 10 ng/ml IFNγ 24 h prior to addition of 0.3 mg/ml ovalbumin. Wells were washed and assayed for cross-presentation 24 h later using reporter cells expressing the OT-I TCR. As the spot numbers after X-gal staining were too numerous to count, the well images were analyzed with the color threshold function of ImageJ to quantify the number of blue pixels. n = 3, ***P<0.001, ****P<0.0001, ANOVA with Bonferroni’s post test. (E) MBECs were analyzed by flow cytometry 24 h after incubation with or without 10 ng/ml IFNγ. Histograms for unstained cells (grey fill), unstimulated cells (dotted line) and IFNγ-stimulated cells (red line) are overlaid. (F) To test whether proteasome inhibitors affected MHC class I expression, IFNγ-stimulated MBECs were incubated with no inhibitor, 10 μM lactacystin, 10 μM MG132 or 100 nM bortezomib for 6 h in the presence of 10−5 ug/ml Pb1 peptide. The wells were then washed and assayed for Pb1 presentation using LR-BSL8.4a cells. As the spots were too numerous to count, the number of blue pixels was quantified as for (D). n = 3, no significant difference by ANOVA. (TIF) [file ppat.1004963.s002.tif]

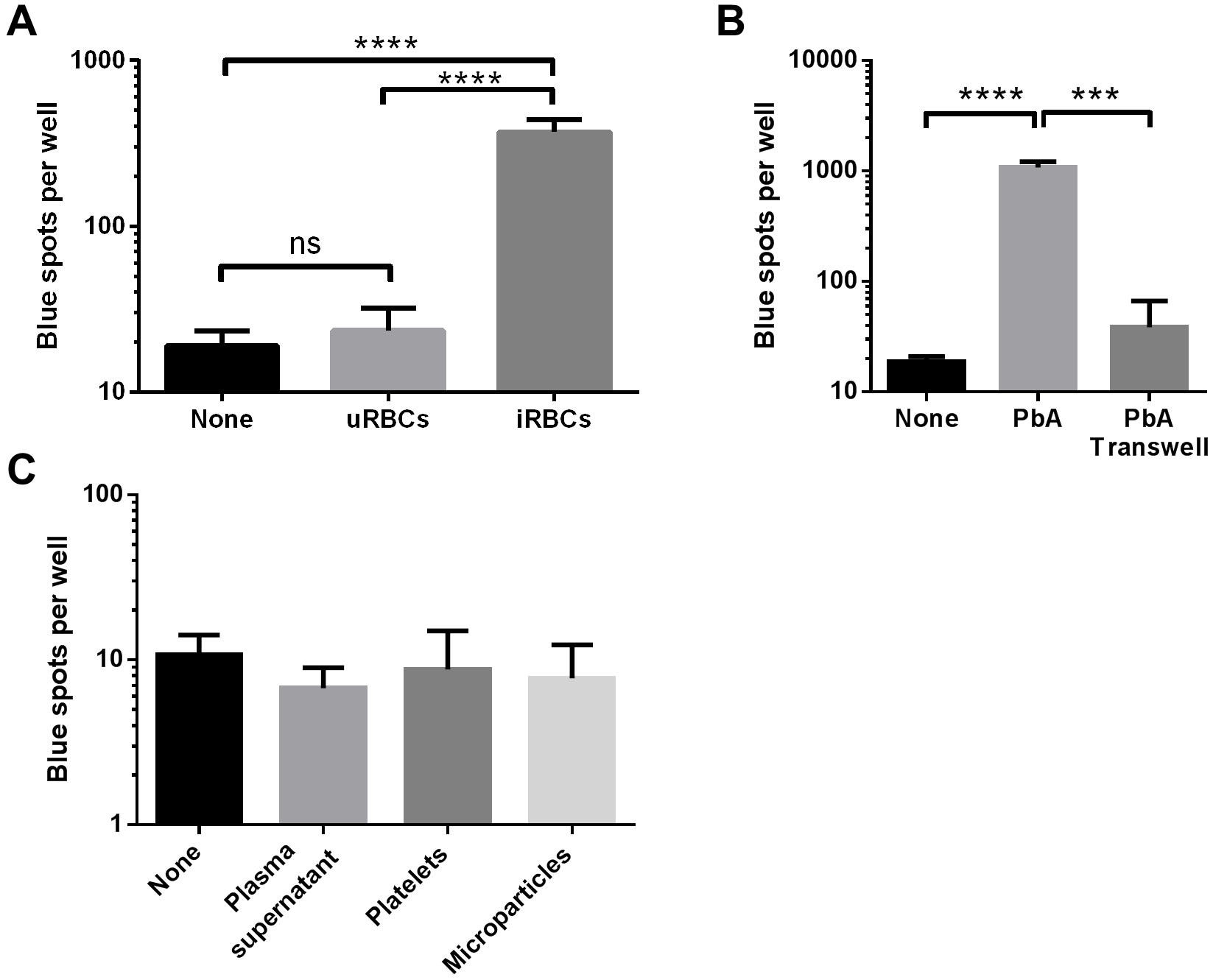

Supplement: S3 Fig — (A) IFNγ-stimulated MBECs were incubated with nothing, 3 × 10^6^ uninfected RBCs (uRBCs) from a naïve mouse or 3×10^6^ PbA mature iRBCs for 24 h, after which cross-presentation of the Pb1 epitope was assayed using LR-BSL8.4a reporter cells. n = 4, ns not significant, ****P<0.0001, ANOVA with Bonferroni’s post test on log-transformed data. (B) MBECs were grown in 24-well plates and stimulated with IFNγ. 6×10^6^ PbA mature iRBCs were added directly to one group of wells. Transwell inserts (0.4 μm pore size) were added to another group of wells, after which the same number of iRBCs were added to the upper chamber. Cross-presentation of Pb1 epitope was assayed after 24 h. n = 3, ***P<0.001, ****P<0.0001, ANOVA with Bonferroni’s post test on log-transformed data. (C) Blood from PbA-infected mice was collected in sodium citrate and sequentially centrifuged at 1500 × g for 15 min (removing RBCs and merozoites), twice at 17 000 × g for 4 min (pelleting the platelets) and at 17 000 × g for 1 h (pelleting the microparticles). Platelets and microparticles coming from 200 μl of plasma were added to wells of IFNγ-stimulated MBECs for 24, after which cross-presentation of Pb1 was measured. n = 4, no significant difference by ANOVA on log-transformed data. (TIF) [file ppat.1004963.s003.tif]

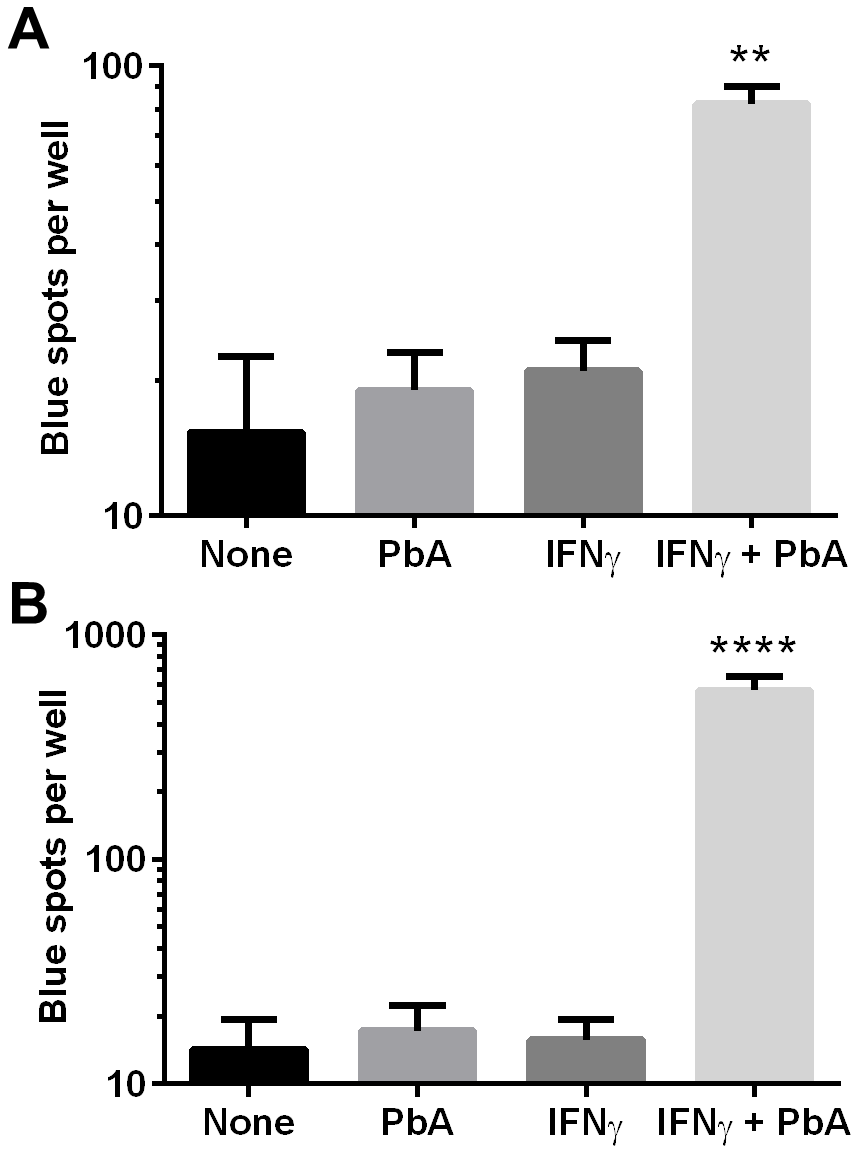

Supplement: S4 Fig — Pericytes were cultured from mouse brain microvessels in two different ways (see below). They were stimulated (or not) with 10 ng/ml IFNγ 24 h prior to addition (or not) of 3 × 10^6^ thawed PbA mature iRBCs. After 24 h, the wells were washed and 6 × 10^4^ LR-BSL8.4a cells were co-incubated overnight, then stained with X-gal. The spot counts were analyzed by ANOVA and Bonferroni’s post test after log transformation. (A) Mouse brain microvessels were cultured in endothelial medium without puromycin selection. When confluent, the cells were detached and sorted for CD45-CD31-NG2+ pericytes, which were seeded in a 48-well plate in complete DMEM medium. The cross-presentation assay was conducted after 2 weeks of growth. n = 3, **P<0.01. (B) Mouse brain microvessels were cultured in endothelial medium without puromycin, passaged once in endothelial medium and twice in Pericyte Medium (ScienCell). There were essentially no CD45+ or CD31+ cells after this. n = 4, ****P<0.0001. (TIF) [file ppat.1004963.s004.tif]
